# Phylogenomic analysis and molecular identification of true fruit flies

**DOI:** 10.3389/fgene.2024.1414074

**Published:** 2024-06-21

**Authors:** Rong He, Shuping Wang, Qiang Li, Zuoqi Wang, Yang Mei, Fei Li

**Affiliations:** ^1^ State Key Laboratory of Rice Biology and Ministry of Agricultural and Rural Affairs Key Laboratory of Molecular Biology of Crop Pathogens and Insects, Institute of Insect Sciences, Zhejiang University, Hangzhou, China; ^2^ Technical Centre for Animal, Plant and Food Inspection and Quarantine, Shanghai Customs, Shanghai, China

**Keywords:** fruit flies, whole genome, phylogenomics, molecular identification, molecular marker

## Abstract

The family Tephritidae in the order Diptera, known as true fruit flies, are agriculturally important insect pests. However, the phylogenetic relationships of true fruit flies, remain controversial. Moreover, rapid identification of important invasive true fruit flies is essential for plant quarantine but is still challenging. To this end, we sequenced the genome of 16 true fruit fly species at coverage of 47–228×. Together with the previously reported genomes of nine species, we reconstructed phylogenetic trees of the Tephritidae using benchmarking universal single-copy ortholog (BUSCO), ultraconserved element (UCE) and anchored hybrid enrichment (AHE) gene sets, respectively. The resulting trees of 50% taxon-occupancy dataset for each marker type were generally congruent at 88% nodes for both concatenation and coalescent analyses. At the subfamily level, both Dacinae and Trypetinae are monophyletic. At the species level, *Bactrocera dorsalis* is more closely related to *Bactrocera latifrons* than *Bactrocera tryoni*. This is inconsistent with previous conclusions based on mitochondrial genes but consistent with recent studies based on nuclear data. By analyzing these genome data, we screened ten pairs of species-specific primers for molecular identification of ten invasive fruit flies, which PCR validated. In summary, our work provides draft genome data of 16 true fruit fly species, addressing the long-standing taxonomic controversies and providing species-specific primers for molecular identification of invasive fruit flies.

## Introduction

The family Tephritidae in the order Diptera, commonly known as true fruit flies, includes over 4,300 species distributed in about 500 genera worldwide (White, 2006). Some species within this family are major agricultural pests globally, threatening various fruits, and causing significant economic losses (Smith et al., 2002). These economically damaging species mainly belong to five genera, *Anastrepha*, *Bactrocera*, *Ceratitis*, *Dacus*, and *Rhagoletis* (Smith et al., 2002).

Though extensive efforts have been devoted to clarifying the phylogeny of fruit flies, the relationships between some groups remain controversial. For example, based on morphological characteristics, the tribe Dacini (*Dacus* + *Bactrocera*) and *Ceratitis* genus belonged to the subfamily Dacinae, and the genera *Anastrepha* and *Rhagoletis* were in the subfamily Trypetinae (Korneyev, 1999). However, molecular evidence does not support the monophyly of Trypetinae (Han and McPheron, 1997; Han and Ro, 2009). Moreover, recent studies have suggested that both the Dacinae and the Trypetinae are non-monophyletic (Krosch et al., 2012; San Jose et al., 2018; Zhang et al., 2019; Song et al., 2019; David et al., 2021; Yong et al., 2021). In the subgenus *Bactrocera*, mitochondrial data shows that *Bactrocera dorsalis* is more closely related to *Bactrocera tryoni* than to *Bactrocera latifrons* (da Costa et al., 2019; Yong et al., 2016; Zhang et al., 2010; Zhang et al., 2018), while nuclear data supports a closer relationship between *B. dorsalis* and *B. latifrons* (Dupuis et al., 2018; San Jose et al., 2018; Valerio et al., 2022). These inconsistences were primarily due to incomplete lineage sorting or introgression (Zhang et al., 2021; Congrains et al., 2023; San Jose et al., 2023). Moreover, most previous molecular studies are mainly based on a few nuclear genes or mitochondrial genome data.

Phylogenetic analyses with a limited number of loci may lead to disputed conclusions (Munro et al., 2011; Young and Gillung, 2020), expanded sets of molecular markers have been used to infer the evolutionary relationships of species across distant taxa (Young and Gillung, 2020). For example, phylogenetic analysis of genome-scale data has tested controversial phylogenetic relationships for a wide range of organisms, such as bacteria (Gomila et al., 2015), fish (Hughes et al., 2018), spiders (Lozano-Fernandez et al., 2019) and asterids (Zhang et al., 2020). Transcriptomes are important genome-scale data widely used for phylogenetic analyses, including Lepidoptera (Bazinet et al., 2017), spiders (Garrison et al., 2016), insects (Misof et al., 2014), Ostracoda (Oakley et al., 2013) and chalcid (Zhang et al., 2020). However, as transcriptomes contain only expressed genes and transcriptome sequencing typically require a large quantity of high-quality RNA (Lemmon and Lemmon, 2013; McCormack et al., 2013), its utility is restricted. In contrast, whole-genome assemblies (Zhang et al., 2019) can obtain near-complete gene sets from a wide range of tissue types. Moreover, it is feasible to sequence the whole genome from low-quality samples such as preserved museum specimens or those intercepted by customs (Huynh et al., 2023). Genome-scale data have been used to infer phylogenies across distant taxa including lice (Boyd et al., 2017), butterflies (Allio et al., 2020), wasps (Cooper et al., 2020), springtails (Sun et al., 2020), and scale insects (Liu et al., 2022). These studies suggest whole genome assemblies are information-rich for phylogenomic analyses.

To explore the genome data for phylogenomic analysis, several types of molecular markers have been developed, including the benchmarking universal single-copy ortholog (BUSCO) gene set (Waterhouse et al., 2018), anchored hybrid enrichment (AHE) (Lemmon et al., 2012), and ultraconserved element (UCE) (Faircloth et al., 2012b; Faircloth, 2016). BUSCO are single copy orthologs identified based on database OrthoDB (Zdobnov et al., 2017) and have been widely used to assess the completeness of genome assemblies and transcriptomes. BUSCO has been used for reconstructing the phylogenies of some organisms, such as yeasts (Shen et al., 2016), insects (Ioannidis et al., 2017), springtails (Sun et al., 2020), and turtles (Gable et al., 2022). In contrast, AHE and UCE target highly conserved regions with variable flanking sequences. AHE gene sets mainly target coding regions, whereas UCE gene sets target coding and noncoding genomic regions (Zhang and Lai, 2020c). AHE has been used to uncover phylogenomic relationships of flower flies (Young et al., 2016), lacewings (Winterton et al., 2018; Cai et al., 2023), beetles (Li et al., 2023) and moths (Homziak et al., 2019). UCE has been used for phylogenetic analysis to recover the relationships of many groups, such as fish (Faircloth et al., 2013), arachnids (Starrett et al., 2017), birds (Stiller et al., 2024), wasps (Cooper et al., 2020), and scale insects (Liu et al., 2022). All these marker sets rely on homology searching in predefined databases or probe sets to identify target loci from genomes (Dietrich et al., 2017; Faircloth, 2017; Breinholt et al., 2018), and have proved useful for inferring phylogenetic relationships at both shallow and deep levels (Zhang and Lai, 2020c; Carter et al., 2023).

Rapid invasive species identification is important for customs departments to develop effective quarantine measures. Presently, the most widely used method for identifying fruit flies relies on the morphological features of adult insects (Huang et al., 2017). However, if intercepted pests are in the stage of larvae or pupae, they need to be reared to adults for accurate identification. This is time-consuming or even impossible to obtain adults because of emergence failure. However, due to the high sequence similarity between true fruit flies, reliable molecular identification primers are still unavailable (Liang et al., 2011; Frey et al., 2013; Manger et al., 2018; Kunprom and Pramual, 2019).

In this study, we used a phylogenomic approach to uncover the phylogeny of Tephritidae to address unclear phylogenetic relationships of true fruit flies. First, genome data of 16 true fruit flies were obtained via Illumina sequencing. Second, we extracted the BUSCO, AHE and UCE from the genome data and built different matrices data to infer the tephritid phylogeny. Moreover, with these genome data, we designed species-specific primers for molecular identification of true fruit flies. Our results provide new insights into the phylogenetic relationship of true fruit flies at the genome level and technical support for quarantine identification of invasive true fruit flies at custom ports.

## Material and methods

### High-throughput sequencing

We collected samples of 16 true fruit fly species for sequencing, across seven genera: *Anastrepha*, *Bactrocera*, *Ceratitis*, *Dacus*, *Zeugodacus*, *Carpomya* and *Rhagoletis* (Table 1). DNA extraction was performed on a single specimen per species, using the Blood and Cell Culture DNA Midi Kit (Qiagen, United States). The quality of genomic DNA was controlled by the following criterion: the concentration of DNA is greater than 30 ng/μL; the OD260/280 range from 1.8 to 2.0; the DNA has no RNA contamination. A 350-bp insert Illumina TruSeq fragment and a 400-bp insert library were constructed from qualified genomic DNA using a TruSeq Nano DNA HT Sample Preparation Kit, and then sequenced on the Illumina Hiseq X-ten and NovaSeq 6000 platforms (see Table 1 for details), respectively. All sequencing data for the 16 fruit flies are available in the National Genomics Data Center GSA database (https://ngdc.cncb.ac.cn/gsa/). The GSA number is CRA016637.

**TABLE 1:** Taxon sampling and genomic information of 25 Tephritidae fruit flies and two *Drosophila* species as outgroups.

No.	Species	Genus	Estimated size (Mb)	Average depth (×)	Contig/Scaffold N50 (Kb)	Contig/scaffold number	Accession number
1	Anastrepha ludens*	*Anastrepha*	1,025	69	10.67	199,750	GWHBPBO00000000
2	Anastrepha suspensa*		1,046	71	9.88	244,021	GWHBPCD00000000
3	Bactrocera correcta**	*Bactrocera*	823	49	15.55	135,326	GWHBPCE00000000
4	*Bactrocera dorsalis*		414	70	1206.00	7,165	GCF000789215.1
5	Bactrocera invadens**		815	55	13.34	121,230	GWHBPBQ00000000
6	*Bactrocera latifrons*		462	101	974.43	3,306	GCF001853355.1
7	*Bactrocera minax*		368	190	94.99	43,124	JAPVRH000000000
8	*Bactrocera oleae*		484	100	4570.89	38,161	GCF001188975.3
9	Bactrocera philippinensis**		677	69	11.39	121,952	GWHBPBS00000000
10	Bactrocera rubigina**		716	60	12.35	121,885	GWHBPBT00000000
11	Bactrocera thailandica**		794	58	12.86	133,684	GWHBPBU00000000
12	*Bactrocera tryoni*		519	96	69.55	31,960	GCA000695345.1
13	Bactrocera tsuneonis*		327	199	96.93	18,487	GWHBPBV00000000
14	Bactrocera zonata**		751	64	16.54	100,872	GWHBPBW00000000
15	Carpomya vesuviana*	*Carpomya*	887	80	21.18	111,305	GWHBPBX00000000
16	*Ceratitis capitata*	*Ceratitis*	471	100	77384.26	71	GCA905071925.1
17	Ceratitis rosa*		1,036	77	3.72	485,326	GWHBPBZ00000000
18	Dacus ciliatus*	*Dacus*	302	228	20.04	35,773	GWHBPBY00000000
19	Dacus punctatifrons*		307	189	56.01	31,835	GWHBPCB00000000
20	Rhagoletis cerasi*	*Rhagoletis*	1,290	47	4.91	493,516	GWHBPCA00000000
21	*Rhagoletis pomonella*		1,223	20	72319.62	32,060	GCF013731165.1
22	*Rhagoletis zephyria*		1,110	35	63.04	84,794	GCF001687245.2
23	*Zeugodacus cucurbitae*	*Zeugodacus*	375	66	1399.02	5,575	GCF000806345.1
24	Zeugodacus scutellata**		646	80	11.75	115,288	GWHBPCF00000000
25	Zeugodacus tau**		653	66	11.72	105,311	GWHBPCC00000000
26	*Drosophila melanogaster*	*Drosophila*	153	21	109.25	5,066	GCA000705575.1
27	*Drosophila novamexicana*		157	10	30.28	16,466	GCA900465405.1

Genomes were sequenced on the Illumina Hiseq Xten platform marked with an asterisk, and Illumina NovaSeq6000 platform marked with two asterisks.

### Genome assembly and annotation

Reads with low-quality bases, adapter sequences, or reads containing poly-Ns were removed using Fastp v0.20.0 (Chen et al., 2018). The reads shorter than 50 bp or with more than 5 Ns were removed, and the reads with the bases whose quality value Q ≤ 15 accounted for more than 50% of total bases were removed. MaSuRCA v3.2.2 (Zimin et al., 2013) with the parameters (GRAPH_KMER_SIZE = auto, USE_LINKING_MATES = 1, LIMIT_JUMP_COVERAGE = 300, CA_PARAMETERS = cgwErrorRate = 0.15, KMER_COUNT_THRESHOLD = 1, and SOAP_ASSEMBLY = 0) was used to assemble the cleaned reads of each species to contig level. All genome assemblies for the 16 fruit flies are available in the National Genomics Data Center GWH database (https://ngdc.cncb.ac.cn/gwh/). All assemblies were assessed for completeness using BUSCOv3.0.2 (Waterhouse et al., 2018) against the Insecta orthodbv9 dataset. Repetitive regions for each genome assembly were masked using Repeat Modeler v2.0.7 (Flynn et al., 2020), and *Ab initio* gene prediction performed in BRAKER v2.1.5 (Bruna et al., 2021) against Arthropoda homology protein dataset (https://bioinf.uni-greifswald.de/bioinf/partitioned_odb11/) following the pipeline by Mei et al. (2022), a combination of automatically training GeneMark-ES/ET/EP v4.59_lic (Lomsadze et al., 2014) and AUGUSTUS v3.3.4 (Stanke et al., 2004).

### Extracting BUSCO, UCE and AHE

For the BUSCO marker, we retrieved the single copy orthologs from the results of each genome assembly by using BUSCO v3.0.2 (Waterhouse et al., 2018) software to scan for the Insecta BUSCO set (1,658 loci).

For UCE and AHE loci, we employed the PHYLUCE v1.6.3 package manual (Faircloth, 2016) to extract UCE and AHE from each genome assembly, using the Diptera-wide UCE2.7kv1 probe set containing 31,328 baits targeting 2,711 loci (Faircloth, 2017) and the Diptera AHE probe set containing 217,702 sites targeting 559 loci (Young et al., 2016), respectively. In the PHYLUCE, the script “phyluce_probe_run_multiple_lastzs_sqlite” was used to align the probe sequence to the assembly genomes. The script “phyluce_probe_slice_sequence_from_genomes” was used to extract the Fasta sequence from the assembly genomes. Then the script “phyluce_assembly_match_contigs_to_probes” was used to match contigs from probes and remove duplicate contigs. Finally, the UCE and AHE loci were extracted using the scripts “phyluce_assembly_get_match_counts” and “phyluce_assembly_get_fastas_from_match_counts.” The flanking region of 400 bp on both sides for each UCE and AHE locus was retained.

### Alignments and matrix generation

For each type of marker, the sequences of each individual locus were aligned with MAFFT v7.475 (Katoh and Standley, 2013), followed by trimming with TRIMAL v1.4.1 (Capella-Gutierrez et al., 2009), and concatenated with FASconCAT-G v1.04 (Kueck and Longo, 2014). Data matrixes of each marker type (BUSCO, AHE) were generated for each locus ensuring at least 50%, 75%, 90%, and 100% species occupancy. Due to an absence of a 100% species-occupancy locus, UCE data matrices were generated for 50%, 75% and 90% taxon-occupancy. Summary statistics were performed using AMAS, including average locus length and parsimony informative sites (Borowiec, 2016).

### Phylogenetic analyses

To infer the phylogenetic relationships of the fruit flies, in addition to the 16 species sequenced in this study, nine previously sequenced fruit fly species from NCBI, including *Zeugodacus cucurbitae*, *B. dorsalis*, *B. latifrons*, *Bactrocera minax*, *Bactrocera oleae*, *B. tryoni*, *Rhagoletis zephyria*, *Rhagoletis pomonella*, *Ceratitis capitata*, and two outgroup species (*Drosophila melanogaster* and *Drosophila novamexicana*) were analyzed. The accession numbers for each species are listed in Table 1. In total, our taxon sampling was 27 taxa including 25 ingroup species and two outgroup species for phylogenetic analyses.

Phylogenomic analyses were conducted using concatenation method, generating supermatrix and coalescent-based species trees for UCE, BUSCO, and AHE matrices. We executed maximum likelihood (ML) of concatenation analysis using partitioning schemes with PartitionFinder v2.1.1 (Lanfear et al., 2017) for the best trees to conduct 20 ML tree searches (10 random and 10 parsimony-based starting trees) and 1,000 bootstrap replicates using RAxML-NG v1.0.1 (Kozlov et al., 2019). For species tree estimation based on the coalescent method, gene trees were first estimated using RAxML-NG v1.0.1 on individual gene alignments with the GTR + G4 substitution model for nucleotides and amino acids with the LG + G4 substitution model by running 500 bootstrap replicates. Species trees were then estimated from gene trees using ASTRAL-III v5.6.1 (Zhang et al., 2018a), using local posterior probabilities to assess node support.

### Calculating Robinson-Foulds (RF) distances

We calculated the pairwise Robinson-Foulds (RF) distances (Robinson and Foulds, 1981) between the topologies of gene trees from BUSCO, UCE, and AHE datasets at 50% species occupancy and their species tree topology using the function *multiRF* in the phytools R package (Revell, 2012). The discordance between all the gene trees and species tree was visualized using a multidimensional scaling method (Duchêne et al., 2018; Roycroft et al., 2020). The pairwise RF distances were plotted in two dimensions using the function *cmdscale* in R and visualized using the ggplot2 package (Villanueva and Chen, 2019).

### Divergence time estimation

Divergence time was computed across each dataset (UCE, BUSCO, and AHE) at 50% species occupancy. We estimated the divergence time utilizing a relaxed molecular clock method using MCMCTree in Paml v4.9 (Yang, 2007). Calibration was performed using three divergence time points obtained from the timetree database (http://timet.ree.org/) and literature. The first calibration point corresponds to the divergence of *Dacus* + *Zeugodacus* (86.3–59.3 Mya) (Krosch et al., 2012). The second calibration point represents the most recent common ancestor of subgenus *Tetradacus* (30.9–12.4 Mya) (Krosch et al., 2012). The final calibration point is the divergence of *Drosophila* (38–62 Mya) (http://timet.ree.org/). To ensure convergence, chains from two independent runs were checked in Tracer 1.7 (Rambaut et al., 2018) to assess the effective sample size (ESS) values above 200, indicating appropriate sampling from the posterior distribution of each parameter. The resulting time trees were viewed and edited using Figtree v.1.4.4 (http://tree.bio.ed.ac.uk/software/figtree/).

### Calculating phylogenetic informativeness

To assess the ability of different marker types to infer relationships at specific time points (Townsend, 2007), the phylogenetic informativeness (PI) of BUSCO, UCE, and AHE nucleotide datasets at 50% species occupancy as measured using TAPIR (Faircloth et al., 2012a), optimized for parallelized calculation across extensive genomic datasets. Before calculating PI, a time-tree was used as an input in this program. We constructed time-calibrated phylogenetic trees for each dataset using our consensus phylogeny topology. The total PI for each dataset and the PI per locus per dataset were calculated, respectively.

### Molecular identification of fruit flies

Considering the availability of the specimens collected, only 13 fruit fly species were used to screen for species-specific primers for molecular identification (Table 2). They were identified by morphological characteristics (Plant Health Australia, 2011; Huang et al., 2017), before the test. Coding sequence (CDS) obtained by *Ab initio* gene prediction of these 13 fruit fly species were blasted against the genome assemblies of all the 25 fruit fly species (Table 1) to predict species-specific sequences by using the following steps: 1) The CDS of each fruit fly was fragmented into 200 bp short sequences with a step length of 50 bp. 2) Then short fragments were searched against genome assemblies for high sequence similarity matches using Bowtie2 v2.5.1 (Langmead and Salzberg, 2012). 3) Fragments with no blast hit in other fruit flies were species-specific. 4) The species-specific fragments were used for designing specific primers and were then verified through PCR.

**TABLE 2:** Information on fruit flies for molecular identification.

No.	Species	Genus	Sex	Life stage	Collection time	Geographic origin of intercept samples	Sample source
1	Anastrepha ludens*	*Anastrepha*	Male	Adult	2015.06	Mexico	Shanghai customs, China
2	Anastrepha suspensa*		Female	Adult	2015.06	Mexico	Shanghai customs, China
3	Bactrocera correcta*	*Bactrocera*	Male	Adult	2016.07	Laos	Shanghai customs, China
4	*Bactrocera dorsalis*		Male	Adult	2018.09	Vietnam	Shanghai customs, China
5	Bactrocera invadens*		Male	Adult	2019.09	India	Guangzhou customs, China
6	*Bactrocera latifrons*		Male	Adult	2018.09	Malaysia	Guangzhou customs, China
7	*Bactrocera minax*		Female	Adult	2016.05	India	Guangzhou customs, China
8	*Bactrocera oleae*		Male	Adult	2017.05	South Africa	Guangzhou customs, China
9	*Ceratitis capitata*	*Ceratitis*	Male	Adult	2016.10	South Africa	Guangzhou customs, China
10	Ceratitis rosa*		Male	Adult	2019.10	United Arab Emirates	Guangzhou customs, China
11	Dacus punctatifrons*	*Dacus*	Male	Adult	2018.01	India	Guangzhou customs, China
12	*Zeugodacus cucurbitae*	*Zeugodacus*	Female	Adult	2020.01	Vietnam	Shanghai customs, China
13	Zeugodacus tau*		Male	Adult	2018.01	Vietnam	Guangzhou customs, China

Protein-coding gene sequences of species were annotated in this study and marked with an asterisk.

Genomic DNA from each species was extracted using the Blood & Cell Culture DNA Midi Kit (Qiagen). The total PCR reaction volume was 20 μL, including 10 μL of PremixTaq (Takara), 1 μL each of primer (10 μM), 1 μL of DNA template, and 7 μL of ddH2O. The PCR reaction consisted of an initial denaturation step at 95°C for 3 min, followed by 35 cycles of denaturation at 94°C for 30 s, annealing at the prime specific temperature (Supplementary Table S4) for 20 s, extension at 72°C for 45 s, and a final extension step at 72°C for 5 min. PCR products were examined via 1.2% agarose gel electrophoresis.

## Results

### Genome assemblies of 16 fruit flies

Each true fruit fly species yielded approximately 30–50 Gb of raw reads, with a sequencing coverage ranging from 47× to 228×. The assembled genome sizes ranged from 302 Mb (*Drosophila ciliates*) to 1,290 Mb (*Rhagoletis cerasi*), with contig N50 lengths spanning from 3.72 (*Ceratitis rosa*) to 96.93 kb (*Bactrocera tsuneonis*), and the number of contigs ranging from 493,516 to 18,487 (Table 1).

**FIGURE 1:**
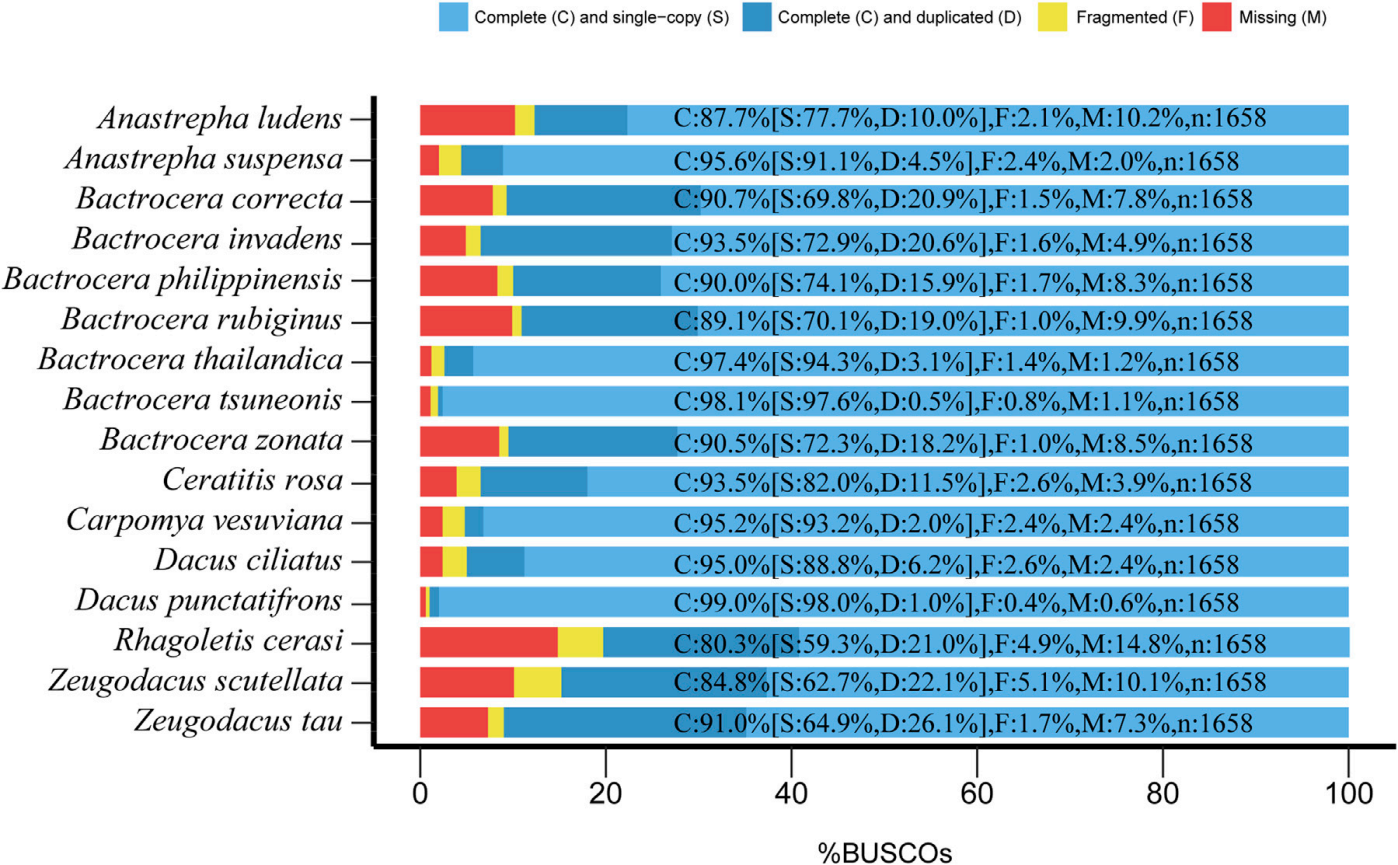
Assessment of genomic completeness of 16 fruit fly species in the Tephritidae family by benchmarking universal single-copy ortholog (BUSCO) analysis using the insect orthoDBv9 dataset containing 1,658 BUSCO genes. C, complete; S, complete single copy; D, complete duplicated; F, fragmented; M, missing. The results showed that 80.3%–99% (1,332–1,650) of BUSCO genes were complete, only 1%–4.9% (2–85) of BUSCO genes were fragmented, and 0.6%–14.8% (6–245) of BUSCO genes were missing, therefore validating these genome assemblies for further analysis.

BUSCO analysis showed that the gene spaces ranged from 85.2% to 99.4% (1,413–1,652 loci). Only 0.6%–14.8% (10–245 loci) were missing (Figure 1), suggesting that these genome assemblies were qualified for subsequent analysis. We used the Braker pipeline (Bruna et al., 2021) to annotate these genomes, yielding a total of 23,046–160,776 protein-coding genes for varied species (Supplementary Table S2).

### Extracting molecular markers of BUSCO, UCE and AHE

We captured phylogenomic data for three loci sets, BUSCO, UCE and AHE, for phylogenetic analysis from the 27 insect genome assemblies. For UCE, *in silico* captured 573–1,842 loci, which ranged from 21% to 68% of the Diptera-wide UCE2.7kv1 probe set (Faircloth, 2017). For AHE, a total of 129–541 loci were extracted, ranging from 23% to 97% of the AHE probe set of Diptera (Young et al., 2016). Relatively more BUSCO loci were extracted (983–1,631), ranging from 59% to 98% of the Insecta orthodbv9 dataset (Supplementary Table S1).

The three molecular markers showed different data matrix patterns. Only a few loci were obtained with 100% presentation. 33 BUSCO loci and one AHE locus were present in all 27 species tested, while s no UCE identified locus was present in all species (Table 3). With the decreasing taxon occupancy, the number of loci in the data matrix for each molecular marker type increased. For BUSCO, an average of 31.52% parsimony informative sites (PIS) of amino acid alignments, and 43.58% PIS of nucleotide alignments were found (50%–100% taxon-occupancy matrix, 1,636–33 loci across 781,442–18,120 amino acid and 2,361,622–55,212 nucleotide sites). For AHE, an average of 36.43% PIS was found in various AHE alignments (50%–100% taxon-occupancy matrix, 135–1 locus across 140,576–1,119 nucleotide sites). For UCE, an average of 44.56% PIS was present in various UCE alignments (50%–90% taxon-occupancy matrix, with 1,327–51 loci across 1,160,133–44,860 nucleotide sites). The percentage of PIS increased as taxon-occupancy was reduced for all three markers (Table 3).

**TABLE 3:** Summary statistics of various datasets.

Types	Matrices	Minimum occupancy per locus (%)	Number of loci	Locus length		Number of sites	Total PIS	Proportion PIS (%)	Missing data (%)
				Mean	Median				
BUSCO	BUSCO50 (AA)	50	1,636	478	388	781,442	239,020	30.60	18.04
	BUSCO50 (NT)	50	1,636	1,444	1,151	2,361,622	1,018,791	43.10	18.03
	BUSCO75 (AA)	75	1,594	481	392	767,872	234,507	30.50	17.62
	BUSCO75 (NT)	75	1,594	1,455	1,163	2,318,678	999,820	43.10	17.56
	BUSCO90 (AA)	90	431	528	411	227,713	72,064	31.60	11.22
	BUSCO90 (NT)	90	431	1,590	1,233	685,214	299,682	43.70	11.05
	BUSCO100 (AA)	100	33	549	412	18,120	6,051	33.40	8.96
	BUSCO100 (NT)	100	33	1,673	1,254	55,212	24,519	44.40	9.10
AHE (NT)	AHE50	50	135	1,041	1,058	140,576	47,895	34.10	36.17
	AHE75	75	72	982	1,024	70,714	25,582	36.20	23.23
	AHE90	90	11	1,038	1,035	11,425	4,125	36.10	11.39
	AHE100	100	1	1,119	1,119	1,119	440	39.30	7.56
UCE (NT)	UCE50	50	1,327	874	918	1,160,133	500,783	43.20	30.06
	UCE75	75	882	867	916	764,504	336,004	44.00	23.74
	UCE90	90	51	880	937	44,860	20,878	46.50	11.54

PIS, AA, and NT mean parsimony informative sites, amino acid, and nucleotide, respectively.

### Recovering phylogenetic relationships of fruit flies

To construct phylogenetic trees for the Tephritidae, we conducted phylogenetic analyses using the different matrices of the three markers (BUSCO, AHE, and UCE). It has been reported that increasing taxon occupancy leads to a reduced loci number (Allio et al., 2020). Increasing the number of loci rather than taxon-occupancy tends to increase phylogenetic tree topological convergence and node support values for each type of marker (Supplementary Figures S1–S8). At 50% taxon-occupancy, the phylogenetic tree topological and node support values for the three types of molecular markers tended to be convergent, based on both concatenation and coalescent methods (Supplementary Figures S1–S8).

**FIGURE 2:**
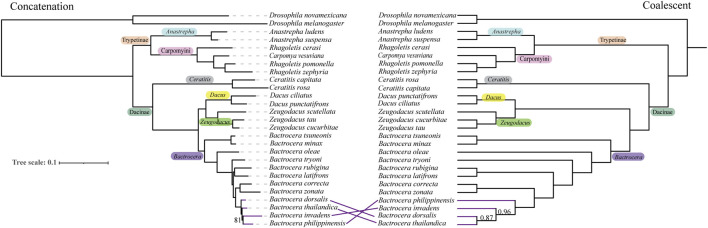
Species trees for fruit flies in the Tephritidae family estimated based on the BUSCO nucleotide matrix of 50% taxon-occupancy amino acid dataset. Concatenation-based RAxML species phylogenetic tree (left) and coalescent-based ASTRAL species phylogenetic tree (right) were inferred by analysis of 1,636 BUSCO loci. Branch support values denote bootstrap support and local posterior probability, respectively. Only support values smaller than 100% or 1 are shown.

The phylogenetic trees inferred using these molecular markers at 50% taxon-occupancy data were generally congruent at most nodes based on both two methods. The family Tephritidae was shown as being comprised of two main clades, Dacinae and Trypetinae with high bootstrap values at the backbone nodes (Figure 2; Supplementary Figures S9–S11). The subfamily Dacinae includes four genera—*Bactrocera*, *Zeugodacus*, *Dacus*, and *Ceratitis*, and the subfamily Trypetinae contains three genera—*Anastrepha*, *Rhagoletis*, and *Carpomya*.

In the Dacinae clade, the genus *Zeugodacus* is sister to the genus *Dacus*, forming a monophyletic group. Within the *Bactrocera* subclade, the subgenus *Bactrocera* forms a monophyletic cluster, separating from the subgenera *Daculus* and *Tetradacus.* In the Trypetinae subfamily clade, the genus *Carpomya* lays close to the paraphyletic cluster *Rhagoletis*, forming a separate subclade from *Anastrepha*. However, all three molecular markers were inconsistent for the *B*. *dorsalis* species complex with ML and ASTRAL analyses (Figure 2; Supplementary Figures S9–S11). The UCE dataset, however, showed a congruent topology based on two different methods (Supplementary Figure S11). *Bactrocera philippinensis* was distant from the other two *Bactrocera* complex species, and *Bactrocera thailandica* was the most closely related to *B. dorsalis* (Supplementary Figure S11). The same result was obtained from the BUSCO dataset based on ASTRAL analysis (Figure 2; Supplementary Figure S9) and the AHE dataset based on ML analysis (Supplementary Figure S10).

### Evaluation of the phylogenetic performance of molecular markers

**FIGURE 3:**
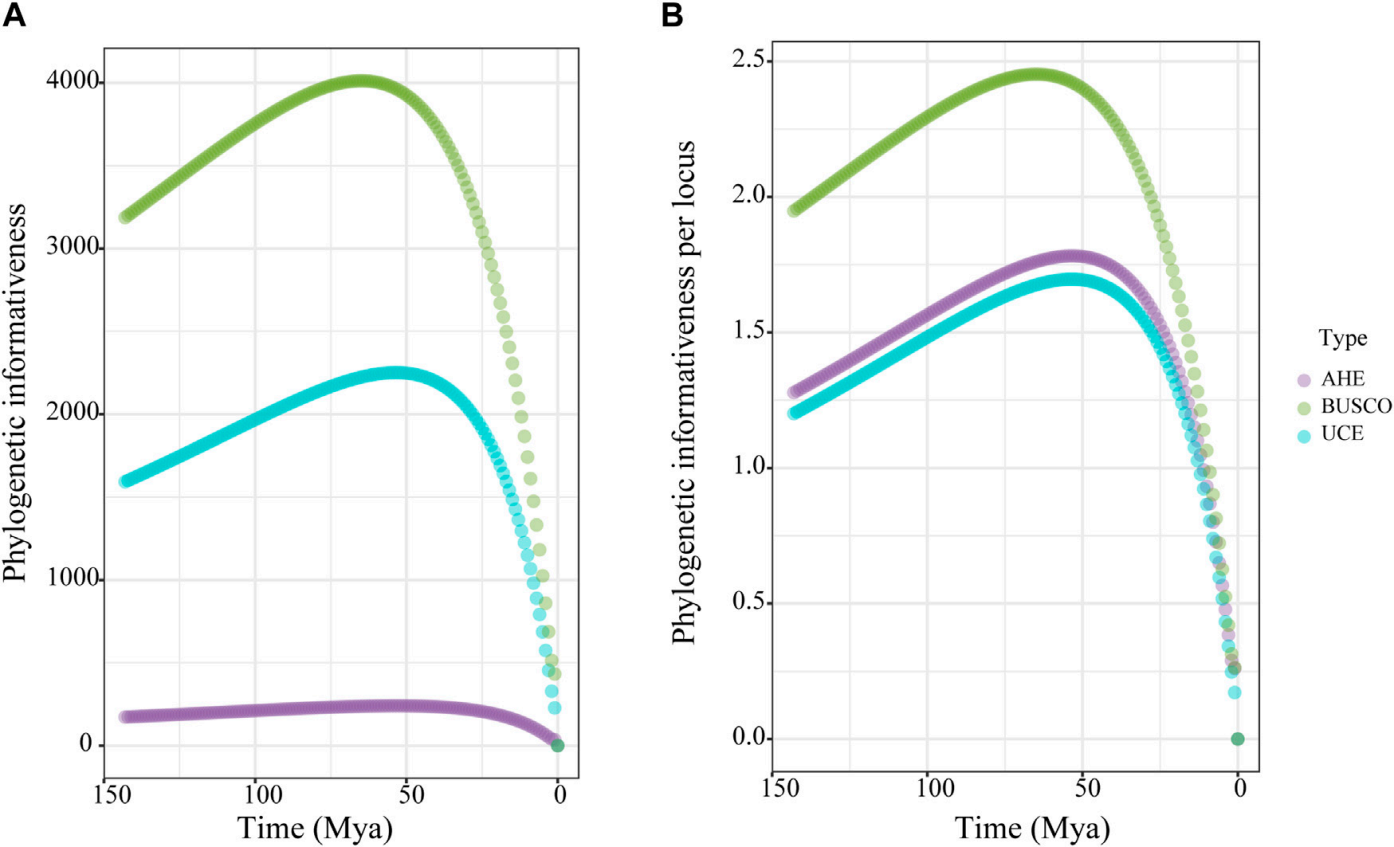
Phylogenetic informativeness (PI) over time for the 50% taxon-occupancy datasets of each molecular marker type. **(A)** total PI, **(B)** PI per locus. Dot means the average PI across all loci of each dataset type for each time point.

To compare the phylogenetic performance of the genomic markers, we used the 50% taxon-occupancy dataset for each marker type. We measured the phylogenetic informativeness (PI) of the three marker types to assess their ability to resolve evolutionary relationships at given time points. The BUSCO dataset showed surpassingly higher total PI than the UCE dataset, both of which were higher than the AHE dataset across all time scales (Figure 3A). For the PI per locus, the three types of markers displayed nearly identical PI over the past 15 Ma. However, the PI value of the BUSCO dataset then rose rapidly and showed higher than both the UCE and AHE datasets from 15 to 150 Ma. During this period, the AHE dataset showed slightly higher PI values than the UCE dataset (Figure 3B). In summary, the PI of our results indicated that the BUSCO dataset contained more robust phylogenetic signals than both UCE and AHE.

**FIGURE 4:**
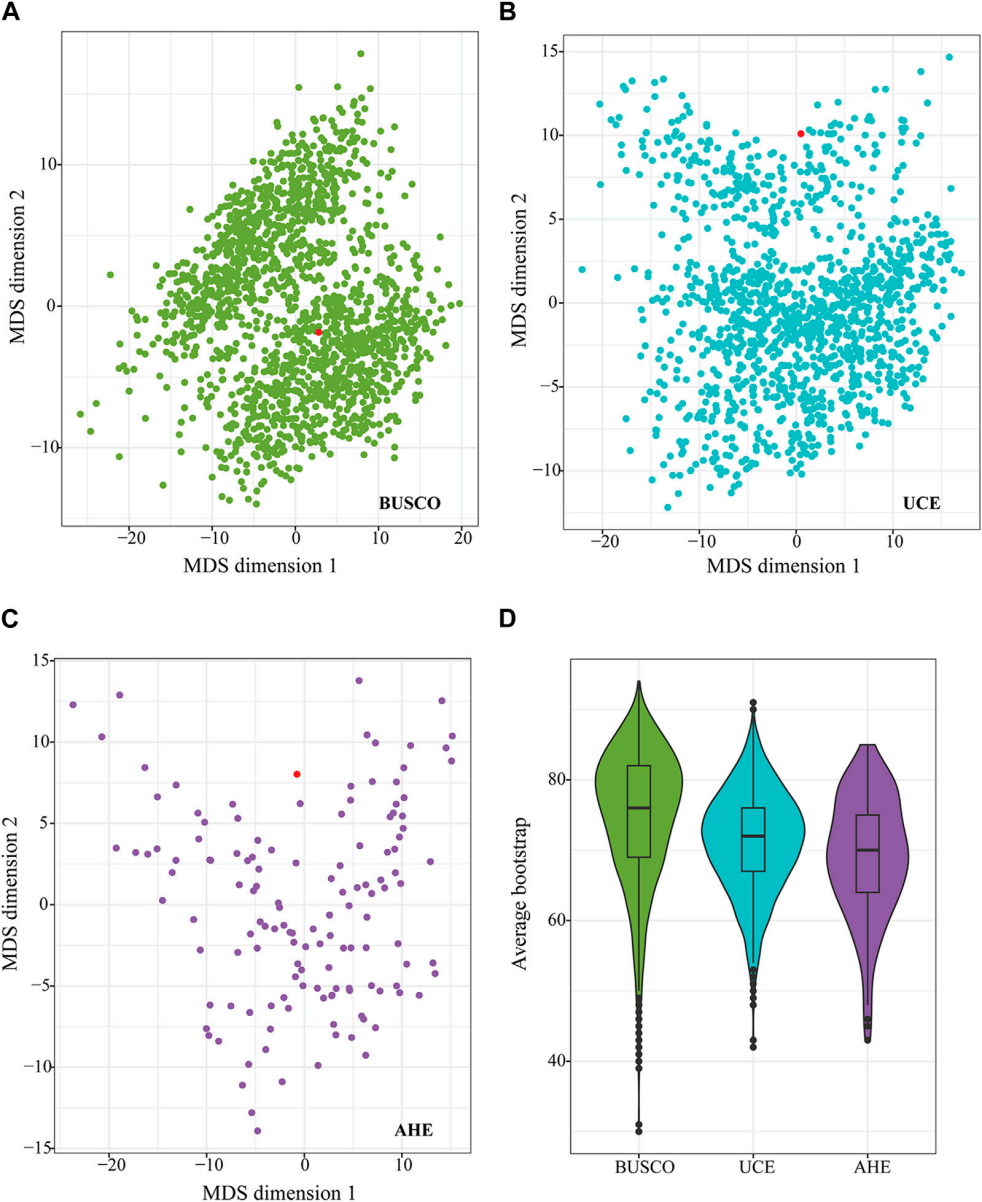
Multidimensional scaling of the pairwise Robinson-Foulds (RF) distance of all gene trees and species trees from BUSCO50 (NT) **(A)**, UCE50 **(B)**, and AHE50 **(C)** datasets. Each dot represents the topology of each gene tree. Distance of pairwise dots represents the RF distance between gene trees. The red dot represents a species tree inferred from the BUSCO50 (NT) dataset using the coalescent method. Average bootstrap values of individual gene trees from each dataset [BUSCO50 (NT), UCE50, and AHE50] are shown in **(D)**.

We also calculated Robinson-Foulds (RF) distances between gene trees topologies from each dataset and species tree topology. Across all marker types, an abundant degree of discordance was observed between the gene tree and species tree (Figures 4A–C). The distribution was scattered and none of the gene trees completely matched the topology of the species trees for each type of marker (Figures 4A–C). The gene trees from the BUSCO dataset, with higher average bootstraps (Figure 4D), most of which were more concentrated, showed less RF distance to the species tree compared to the gene trees from UCE and AHE (Figures 4A–C). In contrast, the gene trees from the AHE dataset were the most scattered among themselves and the species tree, showing the largest degree of difference between gene trees and the species tree (Figure 4C). Therefore, the BUSCO dataset containing more PI exhibited less gene tree heterogeneity and gene tree-species tree heterogeneity and possesses a superior potential to resolve the relationship of the studied true fruit flies.

### The divergence time of the Tephritidae family

**FIGURE 5:**
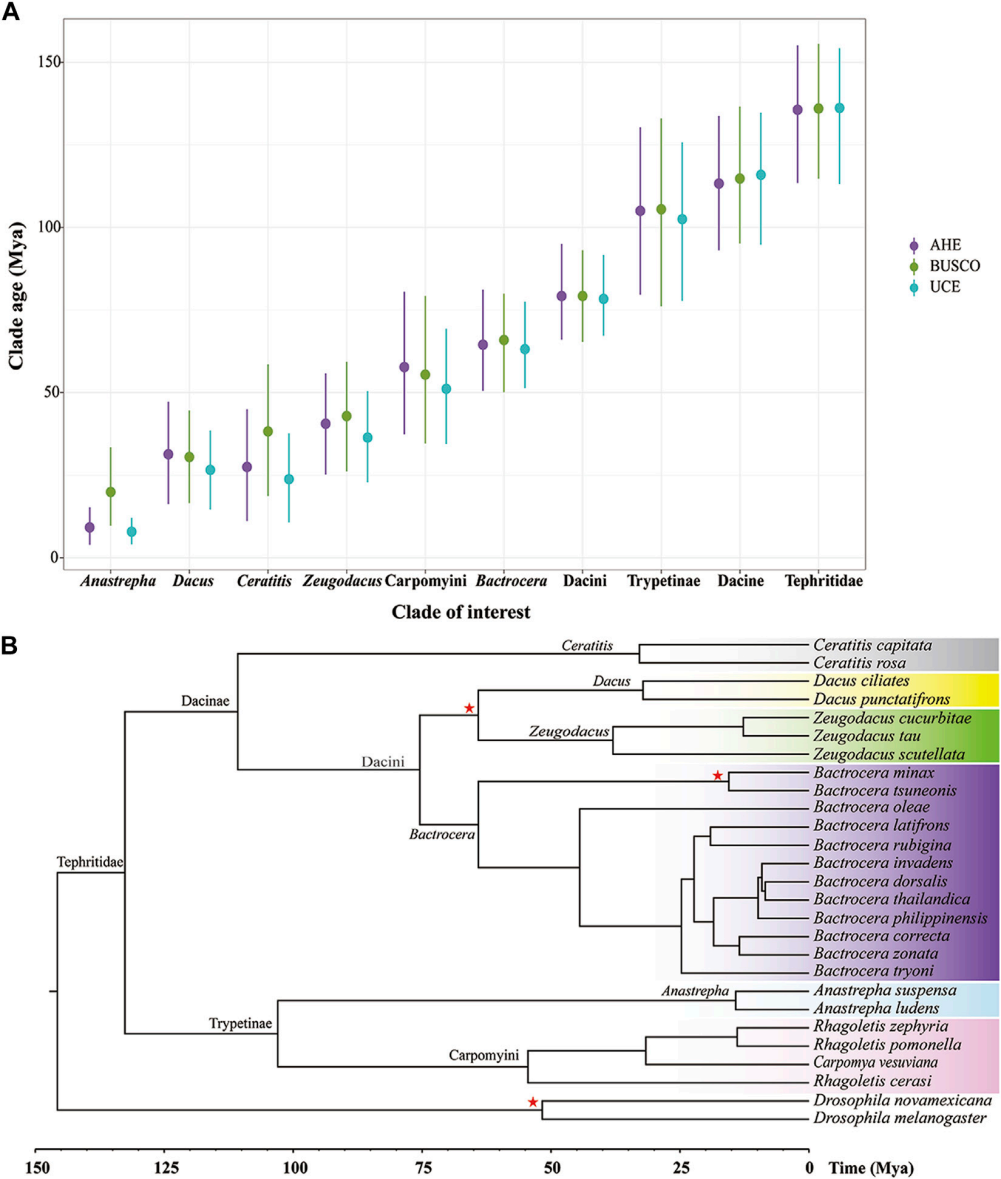
Divergence time of fruit flies in the Tephritidae family estimated by molecular clock analysis. **(A)** Divergence tree estimation of BUSCO50 (NT) dataset performed using MCMCtree. **(B)** Clade age at the genus level and higher level estimated using AHE50, BUSCO50 (NT), and UCE50. Points indicate mean posterior time estimates and lines mean 95% confidence intervals. Red stars: prior calibration. Colors highlight the genus or tribe in the Tephritidae family.

To estimate the divergence time of the Tephritidae family, we used the datasets from UCE50, BUSCO50 (NT), and AHE50. Mean posterior time estimates of all these molecular markers yielded similar results (Figure 5A). However, on the shallower nodes, such as the generic nodes, it seemed that times estimated based on BUSCO tended to be slightly older and feature wider confidence intervals than those based on AHE and UCE, with the youngest age estimates occurring for UCE. In contrast, on the deeper nodes, the estimates from the three markers differed only marginally. This result indicated that time estimates of highly conserved loci were slightly older in the clades that underwent recent rapid radiations.

The MCMC Tree result showed that the crown group of fruit flies (Tephritidae) originated approximately 132.61 Mya on the deepest node, (95% CI: 108.63–153.92 Mya). The subfamilies Dacinae and Trypetinae began to diversify at 110.73 Mya (95% CI: 88.54–132.00 Mya) and 102.98 Ma (95% CI:74.85–131.63 Mya), respectively. The origin of the Dacini tribe and the clade containing *Bactrocera*, *Zeugodacus*, and *Dacus* occurred at 75.44 Mya (95% CI: 63.73–90.16 Mya), 64.09 Mya (95% CI: 50.17–80.59 Mya), 38.00 Mya (95% CI: 21.61–54.24 Mya), and 32.17 Mya (95% CI: 15.78–48.30 Mya), respectively. The *Ceratitis* originated at 32.85 Mya (95% CI: 13.18–54.14 Mya). The most recent common ancestor of the tribe Carpomyini (*Carpomya* + *Rhagoletis*) dated back to 54.47 Mya (95% CI: 32.14–77.91 Mya). The origin of *Anastrepha* occurred at 14.20 Mya (95% CI: 5.36–25.27 Mya) (Figure 5B).

### Molecular identification of fruit flies using species-specific primers

**FIGURE 6:**
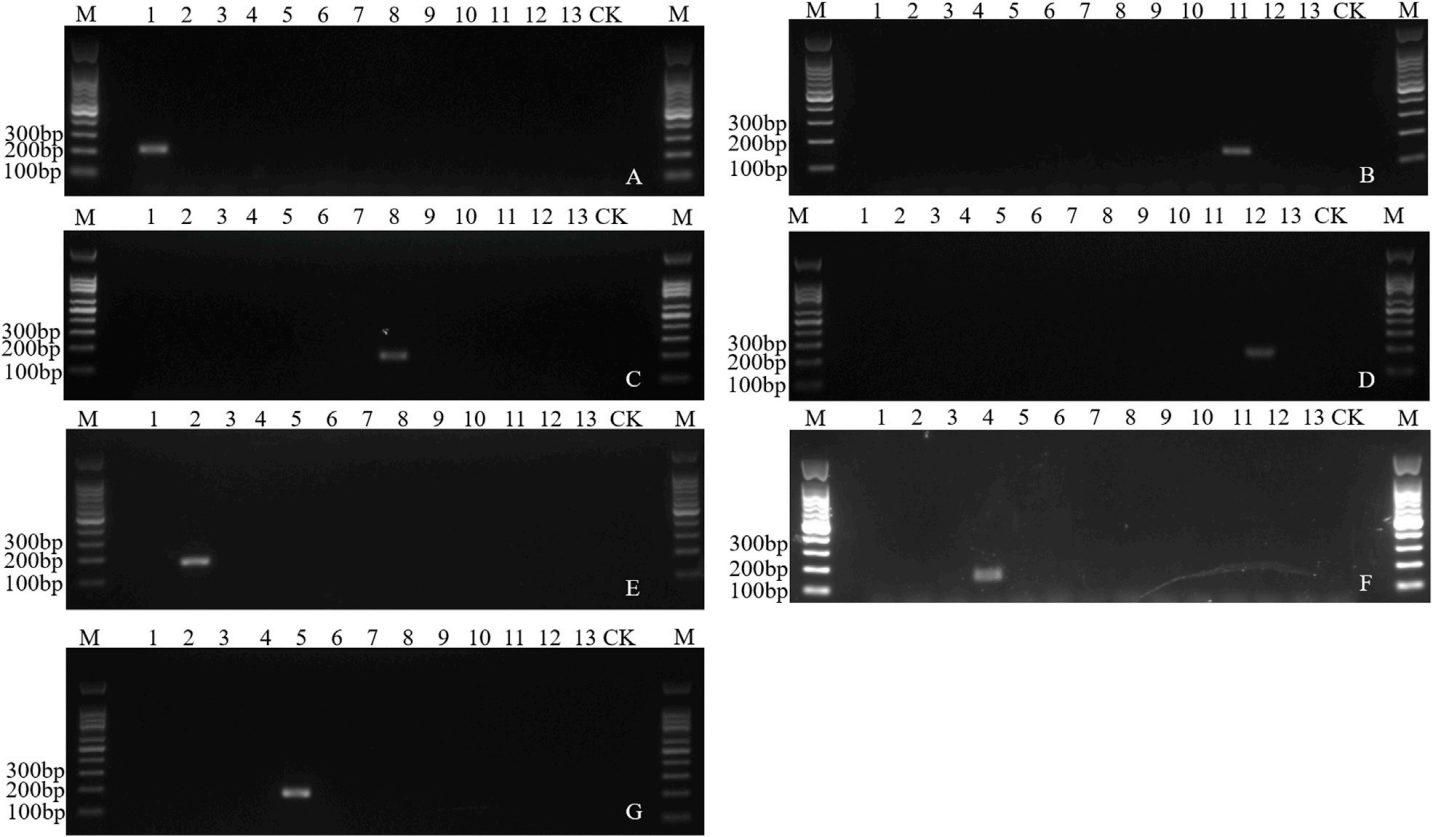
Electropherogram of the PCR amplification of species-specific sequence of fruit flies. **(A)** Specific amplification of Bdor2 in *Bactrocera dorsalis*. **(B)** Specific amplification of Blat4 in *Bactrocera latifrons*. **(C)** Specific amplification of Bole7 in *Bactrocera oleae*. **(D)** Specific amplification of Alud7 in *Anastrepha ludens*. **(E)** Specific amplification of Ztau2 in *Zeugodacus tau*. **(F)** Specific amplification of Zcuc11 in *Zeugodacus cucurbitae*. **(G)** Specific amplification of Ccap2-3 in *Ceratitis capitata*. M: 100 bp DNA marker. Lane 1–13: DNA templates of *Bactrocera dorsalis*, *Zeugodacus tau*, *Bactrocera invadens*, *Zeugodacus cucurbitae*, *Ceratitis capitata*, *Bactrocera minax*, *Bactrocera correcta*, *Bactrocera oleae*, *Dacus punctatifrons*, *Anastrepha suspensa*, *Bactrocera latifrons*, *Anastrepha ludens*, *Ceratitis rosa*. CK, blank control.

The number of species-specific sequences predicted was 4–1,927 among the 13 fruit fly species (Supplementary Table S3). Based on these specific sequences, ten pairs of specific primers, corresponding to ten species, were verified through PCR amplification. The annealing temperature for these primers ranged from 53°C to 60°C, and the product sizes spanned from 101 bp to 184 bp (Supplementary Table S4). A single specific band was found in a total of seven species including *B. dorsalis*, *B. latifrons*, *B. oleae*, *C. capitata*, *Z*. *cucurbitae*, *Zeugodacus tau* and *Anastrepha ludens*, while no amplified fragments were found in other species (Figure 6). Though a single target band was found in *Dacus punctatifrons*, *Bactrocera correcta* and *Anastrepha suspensa*, false negative fragments were also amplified in non-target species, inconsistent with the expected band size (Supplementary Figure S12). Therefore, combined with the amplified fragment size and sequence information, these three species can still be reliably identified. In summary, a total of ten pairs of species-specific primers were screened, which could effectively distinguish ten species from 13 fruit fly species.

## Discussion

The rapidly decreasing sequencing costs have facilitated the fast accumulation of genome data of a wide range of organisms. In contrast to the transcriptome, it is feasible to obtain abundant gene resources from sub-optimal samples such as specimens with 100-year-old history stored in museums (Huynh et al., 2023). The specimens of fruit flies used in this study were intercepted by customs, and the DNA of these samples was usually severely degraded. Although the genome assemblies obtained for these true fruit flies were at the contig level, the BUSCO assessment results showed that the genome completeness of most of them was above 90% (Figure 1), suggesting that these genome assemblies, although fragmented, have a high gene space and are suitable for subsequent phylogenomic analysis.

### Genome-scale data for phylogenetic analysis

To construct a phylogenetic tree with high confidence, we employed different types of molecular markers, namely, BUSCO, UCE and AHE, with varying gene completeness datasets. Extraction proportions for AHE and UCE genes from the genome assemblies of 25 fruit flies ranged from approximately 20%–30% and 30%–50%, respectively, while the extraction proportion for BUSCO genes was above 70% (Supplementary Table S1). However, it should be noted that many genes were absent at 100% species-occupancy for all three types of markers. For instance, UCE was lacking, and only one AHE gene was observed at 100% species occupancy (Table 3). These results suggested a species bias in UCE and AHE, which may be due to the loss of some conserved loci during the genomic evolution of true fruit flies, causing the target loci in the universal Diptera probe set not to be conserved in this rapidly diversifying group (Cohen et al., 2021). Another possible reason is that the evolutionary distances between the studied species and those used for creating probes are too far to find more conserved AHE and UCE loci (Branstetter et al., 2017). For example, the Diptera AHE probe set used in this study was initially designed for flower flies in Syrphidae (Young et al., 2016). One species used for this probe kit was *D. melanogaster*, so it was unexpected that its extraction proportion here was 97% (Supplementary Table S1). However, other species proportions were substantially lower (Supplementary Table S1). Thus, it is necessary to develop a lineage-specific probe set.

### Phylogeny of the fruit flies

Although many studies have addressed the phylogenetic relationship of the Tephritidae family over the past few decades, some controversies remain. Deep level phylogenetic analysis using a limited number of mitochondrial genes, reconfirmed the monophyly of the Dacinae but did not support the non-monophyletic relationship of the Trypetinae, and showed that the tribe Carpomyni (*Rhagoletis + Carpomya*) clustered together with Dacinae rather than *Anastrepha* (Han and McPheron, 1997; Han and Ro, 2009). However, recent studies based on several genes or mitogenome showed that both the Dacinae and Trypetinae are not monophyletic. For instance, the genus *Ceratitis* was closer to the genus *Anastrepha* than to the Dacini tribe (San Jose et al., 2018; David et al., 2021; Yong et al., 2021). In contrast, other studies showed that the *Anastrepha* was closer to the Dacini tribe, forming a distinct cluster from the *Ceratitis* (Krosch et al., 2012; Zhang et al., 2019b; Song et al., 2019). However, our results based on genomic data, showed that *Ceratitis* clustered together with the Dacini tribe and *Anastrepha* clustered together with the Carpomyni tribe, supporting the monophyly of the Dacinae and Trypetinae which aligns with morphological evidence (Korneyev, 1999) (Figure 2; Supplementary Figures S9–S11).

Our results showed that the genus *Zeugodacus* was sister to the genus *Dacus* rather than *Bactrocera*. Morphological evidence regarded *Zeugodacus* as a subgenus of *Bactrocera* (Wang, 1996). However, Krosch et al. (2012) proposed *Zeugodacus* be elevated to the genus level. This was confirmed by subsequent studies using more genes or the mitochondrial genome (Virgilio et al., 2015; San Jose et al., 2018; Zhang et al., 2019b; Yong et al., 2021). Here, we confirmed previous proposals to raise *Zeugodacus* to genus level using whole genome data.

At the shallower levels within the subgenus *Bactrocera*, *B. dorsalis* has generally been regarded as more closely related to *B. tryoni* than to *B. latifrons* which was basal to the subgenus *Bactrocera* based on mitochondrial data (da Costa et al., 2019; Yong et al., 2016; Zhang et al., 2010; Zhang Y. et al., 2018). In contrast, our results showed that *B. dorsalis* was more closely related to *B. latifrons* than to *B. tryoni*, consistent with recent studies based mainly on nuclear data (Dupuis et al., 2018; San Jose et al., 2018; Valerio et al., 2022). Aside from the results obtained by Valerio et al. (2022), those two studies did not conclude the relationships between these three species due to incongruent results from different analysis methods (Dupuis et al., 2018; San Jose et al., 2018). Our results, based on various types of genomic scale datasets with both ML and ASTRAL analyses, supported the closer relationship between *B. dorsalis* and *B. latifrons.* For the *B. dorsalis* species complex, *Bactrocera phillipinensis* and *Bactrocera invadens* were previously considered junior synonyms of *B*. *dorsalis* (Schutze et al., 2015; Schutze et al., 2017). But Drew and Romig proposed the withdrawal of this result (Drew and Romig, 2016). Further evidence based on the male aedeagus showed that *B*. *phillipinensis* and *B*. *invadens* differed from *B*. *dorsalis* (Drew et al., 2022), confirming this withdrawal. Our results show that *B. dorsalis* is more closely related to *B. thailandica* than *B*. *phillipinensis* and *B*. *invadens* (Figure 2; Supplementary Figures S9–S11).

### Molecular identification of fruit flies

Molecular identification is not limited to the insect stage and specimen integrity, which is a simple and accurate method. DNA barcoding based on the mitochondrial cytochrome oxidase I gene (COI) (Hebert et al., 2003) has been widely used in species identification (Hajibabaei et al., 2006; Nopparat et al., 2010). However, many problems have emerged, such as the close genetic distance between species, sequence similarity, and the interference of mitochondrial pseudogenes, resulting in an inability of COI to accurately distinguish between the species of fruit flies (Liang et al., 2011; Blacket et al., 2012; Manger et al., 2018). Compared to DNA barcoding, which uses a single gene for identifying species, large numbers of potential candidate diagnostic loci were quickly obtained from whole genome assemblies in this study (Supplementary Table S3). These alternative diagnostic loci may circumvent the above-mentioned issues and provide a greater range of tools for species identification. Furthermore, the species-specific identification method used here has the advantages of speed and cost-effectiveness, unlike the tree-based COI diagnostics methods. In recent years, species-specific simple repeat sequences from the genome were successfully used for the molecular identification of four fruit fly species including *C. capitata*, *Z*. *cucurbitae*, *B. dorsalis* and *B. tryoni* (Ding et al., 2018). However, simple repeat sequences are usually located in non-coding regions, and there are large differences in repeat sequences between individuals of the same species (Miah et al., 2013), making it challenging to ensure their stability in amplification. The CDS used to screen species-specific sequences in this study, encode protein products and are relatively stable in PCR amplification. The PCR products are also relatively easy and stable to amplify due to their size, ranging from 100 to 200 bp (Supplementary Table S4), conducive to the repetition in molecular identification. False negative amplification has also been observed in other non-target species. Moreover, although the species-specific markers such as Bcor7, Ztau2, and Dpun6, had no hits in the NT database using BlastN, they showed some extent of similarity with bacterial proteins using BlastX. Whether these markers are reliable for effective molecular identification needs further verification. Therefore, more samples are required to verify the selected specific primers in the future. We successfully screened ten pairs of specific primers corresponding to ten species based on a broad survey of whole genome assemblies. Our results provided technical support for the quarantine inspection of invasive fruit flies while enriching the gene resources for identifying fruit flies and presenting new ideas for molecular diagnostic marker screening.

## Data Availability

The datasets presented in this study can be found in online repositories. The names of the repository/repositories and accession number(s) can be found in the article/Supplementary Material.
