# Supplementary material for: Phylogenomic analysis and molecular identification of true fruit flies
Source: Front Genet. 2024 Jun 21;15:1414074. doi: 10.3389/fgene.2024.1414074 (PMC11224437; doi:10.3389/fgene.2024.1414074)
Supplement: Supplementary file 1 [file DataSheet1.docx]

**Table S1. Loci number per species for the three markers**.

| **No.** | | **Species** | **BUSCO** | | **AHE** | | **UCE** | |
| --- | --- | --- | --- | --- | --- | --- | --- | --- |
|  |  |  | **Total number** | **Extraction proportion**  **(%)** | **Total number** | **Extraction proportion**  **(%)** | **Total number** | **Extractionproportion(%)** |
| 1 | *Anastrepha_ludens* | | 1,288 | 78 | 129 | 23 | 1,064 | 39 |
| 2 | *Anastrepha suspensa* | | 1,511 | 91 | 145 | 26 | 1,266 | 47 |
| 3 | *Bactrocera correcta* | | 1,158 | 70 | 169 | 30 | 934 | 34 |
| 4 | *Bactrocera dorsalis* | | 1,590 | 96 | 169 | 30 | 1,393 | 51 |
| 5 | *Bactrocera invadens* | | 1,209 | 73 | 146 | 26 | 793 | 29 |
| 6 | *Bactrocera latifrons* | | 1,625 | 98 | 167 | 30 | 1,422 | 52 |
| 7 | *Bactrocera minax* | | 1,616 | 97 | 169 | 30 | 1,356 | 50 |
| 8 | *Bactrocera oleae* | | 1,631 | 98 | 163 | 29 | 1,397 | 52 |
| 9 | *Bactrocera philippinensis* | | 1,228 | 74 | 154 | 28 | 919 | 34 |
| 10 | *Bactrocera rubiginus* | | 1,162 | 70 | 144 | 26 | 918 | 34 |
| 11 | *Bactrocera thailandica* | | 1,563 | 94 | 154 | 28 | 1,199 | 44 |
| 12 | *Bactrocera tryoni* | | 1,544 | 93 | 169 | 30 | 1,370 | 51 |
| 13 | *Bactrocera tsuneonis* | | 1,619 | 98 | 162 | 29 | 1,409 | 52 |
| 14 | *Bactrocera zonata* | | 1,198 | 72 | 144 | 26 | 840 | 31 |
| 15 | *Carpomya vesuviana* | | 1,545 | 93 | 141 | 25 | 1,311 | 48 |
| 16 | *Ceratitis capitate* | | 1,579 | 95 | 190 | 34 | 1,396 | 51 |
| 17 | *Ceratitis rosa* | | 1,360 | 82 | 150 | 27 | 829 | 31 |
| 18 | *Dacus ciliates* | | 1,473 | 89 | 161 | 29 | 1,184 | 44 |
| 19 | *Dacus punctatifrons* | | 1,625 | 98 | 177 | 32 | 1,347 | 50 |
| 20 | *Rhagoletis cerasi* | | 983 | 59 | 130 | 23 | 841 | 31 |
| 21 | *Rhagoletis pomonella* | | 1,327 | 80 | 135 | 24 | 1,042 | 38 |
| 22 | *Rhagoletis zephyria* | | 1,296 | 78 | 145 | 26 | 573 | 21 |
| 23 | *Zeugodacus cucurbitae* | | 1,639 | 99 | 184 | 33 | 1,428 | 53 |
| 24 | *Zeugodacus scutellata* | | 1,039 | 63 | 151 | 27 | 845 | 31 |
| 25 | *Zeugodacus tau* | | 1,076 | 65 | 153 | 27 | 810 | 30 |
| 26 | *Drosophila melanogaster* | | 1,607 | 97 | 541 | 97 | 1,842 | 68 |
| 27 | *Drosophila novamexicana* | | 1,631 | 98 | 226 | 40 | 1,686 | 62 |

**Table S2. Statistics of gene annotation of 16 species of fruit flies**

| Species | Annotation of gene | | | | |
| --- | --- | --- | --- | --- | --- |
|  | Number | Busco assessment （n=1658）% | | | |
|  |  | S | D | F | M |
| *Anastrepha ludens* | 41,077 | 74.8 | 11.6 | 3.0 | 10.6 |
| *Anastrepha suspensa* | 48,148 | 89.6 | 5.2 | 2.7 | 2.5 |
| *Bactrocera correcta* | 52,728 | 65.9 | 23.5 | 2.4 | 8.2 |
| *Bactrocera invadens* | 80,819 | 63.6 | 20.6 | 2.2 | 13.6 |
| *Bactrocera philippinensis* | 63,125 | 71.2 | 17.0 | 1.7 | 10.1 |
| *Bactrocera rubigina* | 52,578 | 66.9 | 20.9 | 2.0 | 10.2 |
| *Bactrocera thailandica* | 87,547 | 88.9 | 3.7 | 1.3 | 6.1 |
| *Bactrocera tsuneonis* | 23,046 | 95.3 | 0.9 | 1.2 | 2.6 |
| *Bactrocera zonata* | 59,105 | 68.3 | 19.8 | 1.7 | 10.2 |
| *Carpomya vesuviana* | 51,273 | 70.9 | 7.3 | 3.7 | 18.1 |
| *Ceratitis rosa* | 160,776 | 67.6 | 15.9 | 2.7 | 13.8 |
| *Dacus ciliatus* | 47,373 | 70.8 | 6.6 | 4.0 | 18.6 |
| *Dacus punctatifrons* | 58,278 | 84.6 | 1.9 | 1.4 | 12.1 |
| *Rhagoletis cerasi* | 53,331 | 56.9 | 23.3 | 5.3 | 14.5 |
| *Zeugodacus tau* | 83,074 | 62.1 | 28.0 | 1.9 | 8.0 |
| *Zeugodacus scutellata* | 133,807 | 57.8 | 25.2 | 5.4 | 11.6 |

C: complete and single-copy genes, D: complete and duplicated genes, F: fragmented genes, M: missing genes.

**Table S3. Results of species-specific sequences in thirteen fruit fly species**

| No. | Species | Number of specific sequences |
| --- | --- | --- |
| 1 | *Anastrepha ludens* | 41 |
| 2 | *Anastrepha suspensa* | 187 |
| 3 | *Bactrocera correcta* | 35 |
| 4 | *Bactrocera dorsalis* | 4 |
| 5 | *Bactrocera invadens* | 1,046 |
| 6 | *Bactrocera latifrons* | 51 |
| 7 | *Bactrocera minax* | 83 |
| 8 | *Bactrocera oleae* | 21 |
| 9 | *Ceratitis capitate* | 263 |
| 10 | *Ceratitis rosa* | 1,927 |
| 11 | *Dacus punctatifrons* | 1,092 |
| 12 | *Zeugodacus cucurbitae* | 183 |
| 13 | *Zeugodacus tau* | 109 |

**Table S4. Species specific primers based on the species-specific sequence**

| Species | Sequence number | Species-specific sequence | Specific primer | Annealing  Temperature (℃) | Product length  (bp) |
| --- | --- | --- | --- | --- | --- |
| *Anastrepha ludens* | Alud7 | CAAAGAAACCAACTACAAATGATATCTCTGAACCCAGCCCTAAAAGTGCTGCTGTGGAGCCTATACGAAATAGTAATGCAGACCTACTATCGGATTTACTTGGTAACGAATCAAATATAACGCCGACCACGGAGTCGGCTAATGCTGCGTCGTCCATTGATGAAGTGGAGTTACTGGGTGCAACTGCTAATTTGCCTACC | F: TCTCTGAACCCAGCCCTAAA  R: TGCACCCAGTAACTCCACTTC | 56 | 158 |
| *Anastrepha suspensa* | Asus3 | ATGAGTAGCATTTTTTTCATCCGCCCAAACAGAATTCTACACTCTTACAAAGCCTGTAGAAATTCAAAGCTTACACGTCTCCTCCATTCTTCAGAAAAAATAATTCCTATCCGCAATTCGAAATCCCTACGTTTTCGCTCTTACTTTGGAATCGGCTTACTTGGAGTGTTTACAGGTTTTGTTGCGTATGATGGAGTTGT | F: CGCCCAAACAGAATTCTACAC  R: CCATCATACGCAACAAAACCT | 54 | 173 |
| *Bactrocera correcta* | Bcor7 | CTCAATGGCGGCAGCTTGCTCGACTATGACCTGATCGAGGGGCAGCTGGTTTCGCCCTTCGGCCAGTTCCGGGGCCTGTCGGAAGAGCCGGACGCCGGCTTCGGGCGCACGGTCGAGCGCGACGAGGCGGGCAAGCGCATGGCGGCCTATGAGCCGGGGCAGTTCGTCCGGTTCGTGCCGCGCCGGCTCGACGGGCTGAC | F: GCTTGCTCGACTATGACCTG  R: GGCACGAACCGGACGAAC | 56 | 167 |
| *Bactrocera dorsalis* | Bdor2 | AGTAGTTGTGATGGCGTTTTGATCGAGAAACGTTTTGTGTTAATTGCACGAATGGCCTATAATATTGAACAACAAAAAATACTCAATTTTTTTTTAACTTATAGATATAAGGCAGTGTCCAAGAAAATAAAGAAAGTACATACCCTAAGAACGTTTATGCTTGAATATATTGCTTTGGTTGAACTGGATAGTGATGTGGA | F: TTAATTGCACGAATGGCCTA  R: ATCCAGTTCAACCAAAGCAA | 58 | 150 |
| *Bactrocera latifrons* | Blat4 | ACAGCGTTAATAGTGAAGATGAACGTCGTGATAAACGCCAGAGTAAGCAACGAGCAACTTATAACGATGACAGCAATAAGCATAGACGATCCACTGTAGAGGCTCATAAAAAAATATTAAACAGAGATAAAAGCCGAAGCCGCTCAAAATACAAGAGATAA | F: ACGCCAGAGTAAGCAACGAG  R: CGGCTTCGGCTTTTATCTCT | 58 | 109 |
| *Bactrocera oleae* | Bole7 | ATATACGACTGCTATTGGCTTCTCAGCTCCGCAGGTGCCAAATATCGGTTCACCGTTGGCATTTCCAGCAGCACCTATAGCAACCACATTTGCGGCGCCAAATACAGCTTATGGTGCTCCGGTGGCATATGCTGCTCCACCATCTTTCTTAGGTTCAGCAAGGTATGCCGCACCCACAGTGCTCTCCACAGGATCTCTGT | F: GGTGCCAAATATCGGTTCAC  R: CAGAGATCCTGTGGAGAGCA | 56 | 101 |
| *Ceratitis capitate* | Ccap2-3 | TTAGCGTTTCTCTGGACGATAACGCATTTGTGGAAAATCCTAATAAAGAAAGCGATATTTCGGAAAGTTTCAATGACGATGCTGAAGAATTCGAATCAGATAAAGAACCAACTACTAGTACACGCAGGTACAATGTTCAACCCGCTAAGTGTGAAACGTGTGGAAAAGTTTTTAAAAACAAGTCGCGTTTACGCAGGCAC | F: TGGACGATAACGCATTTGTGG  R: CTGCGTAAACGCGACTTGTT | 56 | 184 |
| *Dacus punctatifrons* | Dpun6 | AGACTATGCTTGGTCATGGCGCGGCCTTGGAAACGGTCATCATGAAAATTGTCGGAAAAACTGCAGCAGACATCTTTGACAGCATCCGCATGCAGACCCAGGCTGGCGTGCTGCAGCCGGGGCAGGCGTTGCCAACCGTGCGCGACCTGGCCGTCACGCTGGAGGTCAACCGCAATACCATATCGATGGCCTATCAGCGC | F: TGGAAACGGTCATCATGAAA  R: TCGATATGGTATTGCGGTTG | 53 | 139 |
| *Zeugodacus cucurbitae* | Zcuc11 | TTTCTTCAAAAGATTCTTGAAGAAGATAACGCAAAAAGTGAAAAAGATATTCTAGGTTCCTTTGATCGCGATGTCCTTGAAGAAGAAGAGAAAGAATTGGATCACTTTCCTTCAAGCATGACTGAAGAAGACTCCACAGATAACAATAGCTCAAAGCAGGAACAAGAATTCGAAAATATTCTTCAAAAGATCCTTGAAGA | F: GTTCCTTTGATCGCGATGTC  R: TTCTTGTTCCTGCTTTGAGC | 60 | 113 |
| *Zeugodacus tau* | Ztau2 | TACCACGAATTACTACCTGAAGCGCCAAAGATTCGGGCGCTGAATGACAGGCGTAAGAACGCGATTCAAACGTTCTGGCGGAAAGCTGGAGTAATCACCCGGCAACTGGACGGCCATGGGTTCACCCTGGAAGACTGGAAATCTTATCTGAGCTATGTAGCGACAAATTGCCGCTGGATGTTCGAAGAGCGCACCAATCA | F: TACCTGAAGCGCCAAAGATT  R: ACATCCAGCGGCAATTTGT | 58 | 168 |

F: forward primer; R: reverse primer


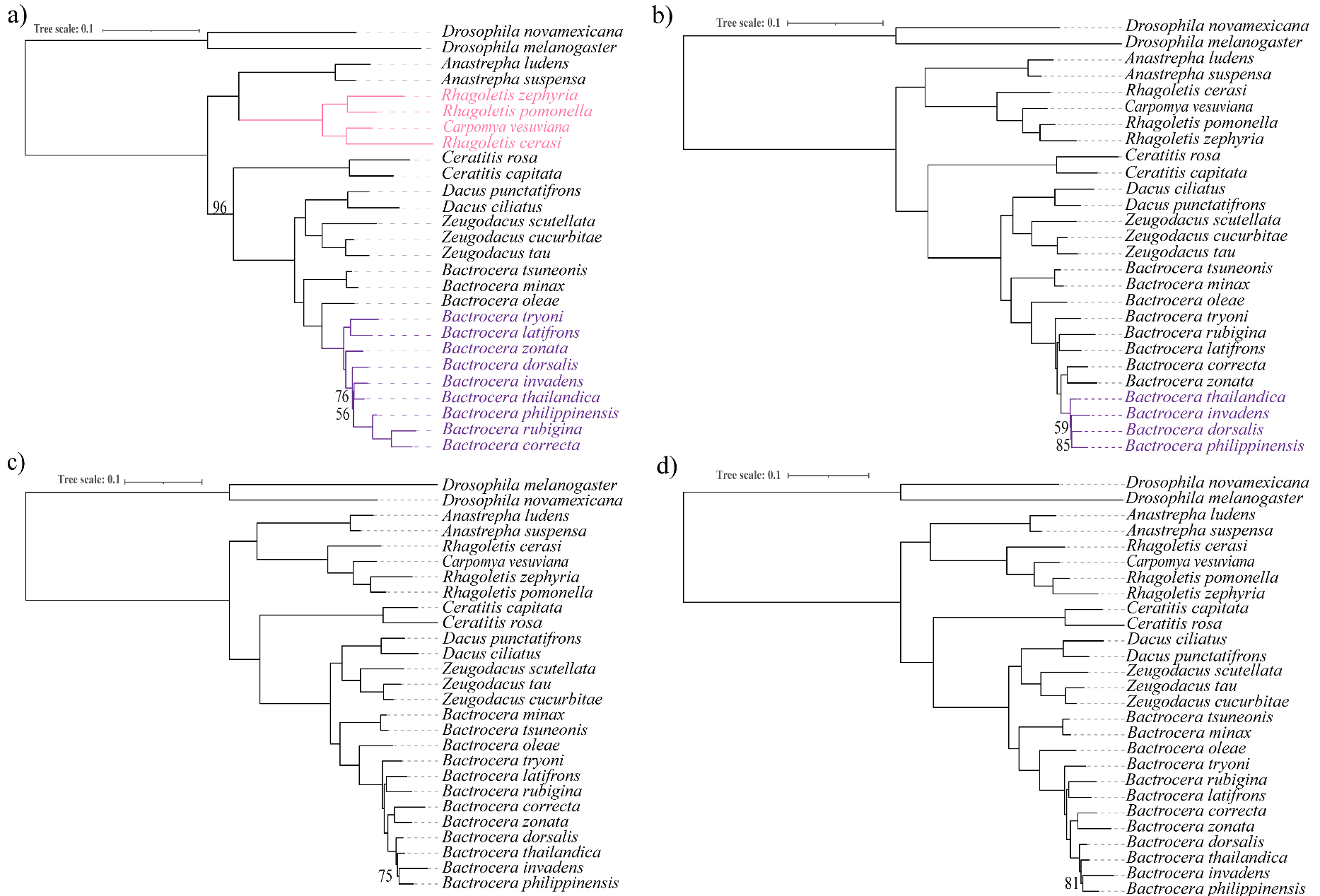


**Figture S1. Phylogeny tree of fruit flies estimated for BUSCO nucleotide matrix of different taxon-occupancy datasets using a concatenation-based method.** a) 100% taxon-occupancy BUSCO nucleotide dataset, b) 90% taxon-occupancy BUSCO nucleotide dataset, c) 75% taxon-occupancy BUSCO nucleotide dataset, d) 50% taxon-occupancy BUSCO nucleotide dataset. Increasing the number of loci tended to increase phylogenetic tree topological convergence and node support values. Colors highlight unstable topologies. Only support values smaller than 100 are shown.


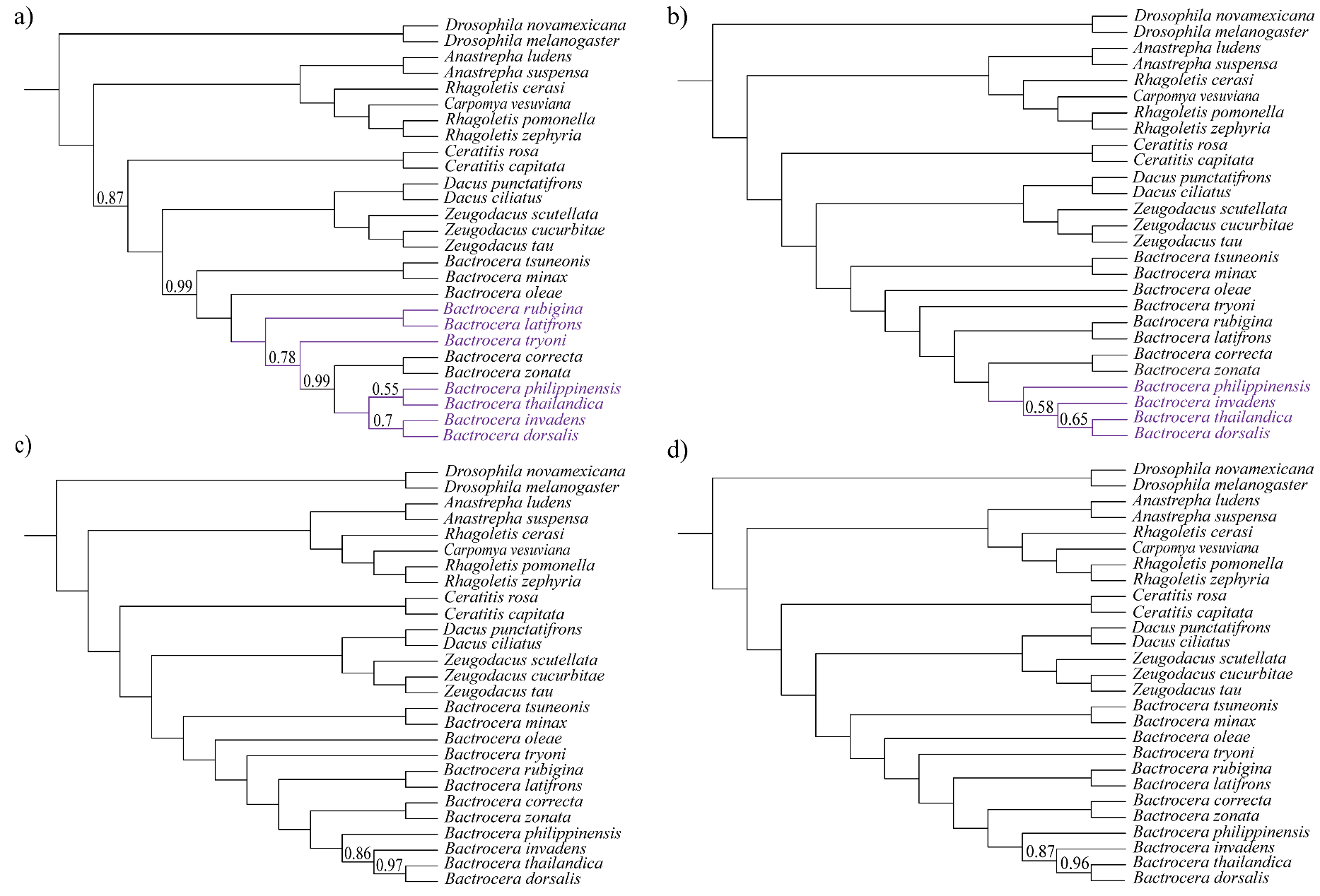


**Figture S2. Phylogeny tree of fruit flies estimated for BUSCO nucleotide matrix of different taxon-occupancy datasets using a coalescent-based method.**

a) 100% taxon-occupancy BUSCO nucleotide dataset, b) 90% taxon-occupancy BUSCO nucleotide dataset, c) 75% taxon-occupancy BUSCO nucleotide dataset, d) 50% taxon-occupancy BUSCO nucleotide dataset. Increasing the number of loci tended to increase phylogenetic tree topological convergence and node support values. Colors highlight unstable topologies. Only support values smaller than 1 are shown.


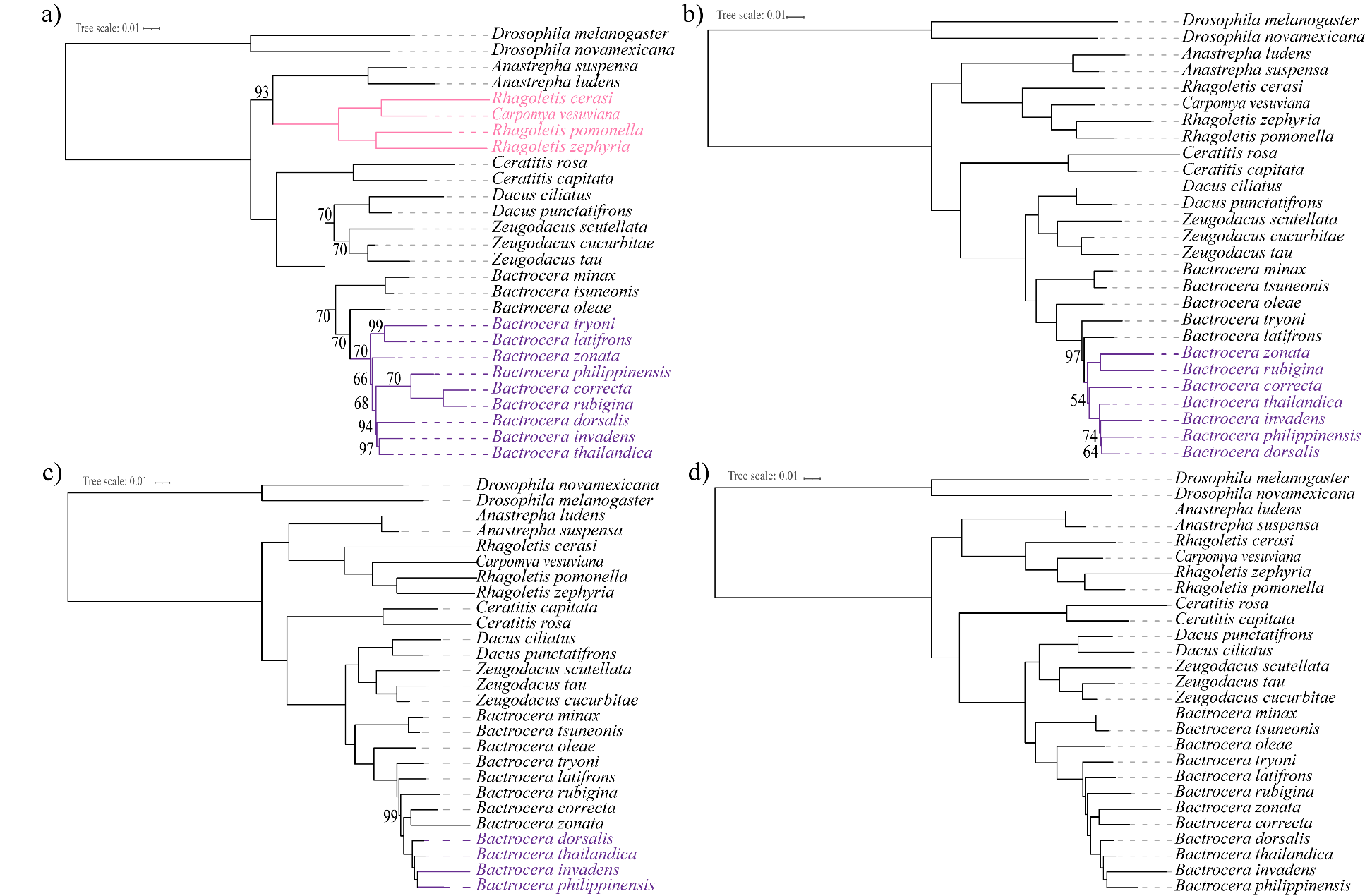


**Figture S3. Phylogeny tree of fruit flies estimated for BUSCO amino acid matrix of different taxon-occupancy datasets using a concatenation-based method.**

a) 100% taxon-occupancy BUSCO amino acid dataset, b) 90% taxon-occupancy BUSCO amino acid dataset, c) 75% taxon-occupancy BUSCO amino acid dataset, d) 50% taxon-occupancy BUSCO amino acid dataset. Increasing the number of loci tended to increase phylogenetic tree topological convergence and node support values. Colors highlight unstable topologies. Only support values smaller than 100 are shown.


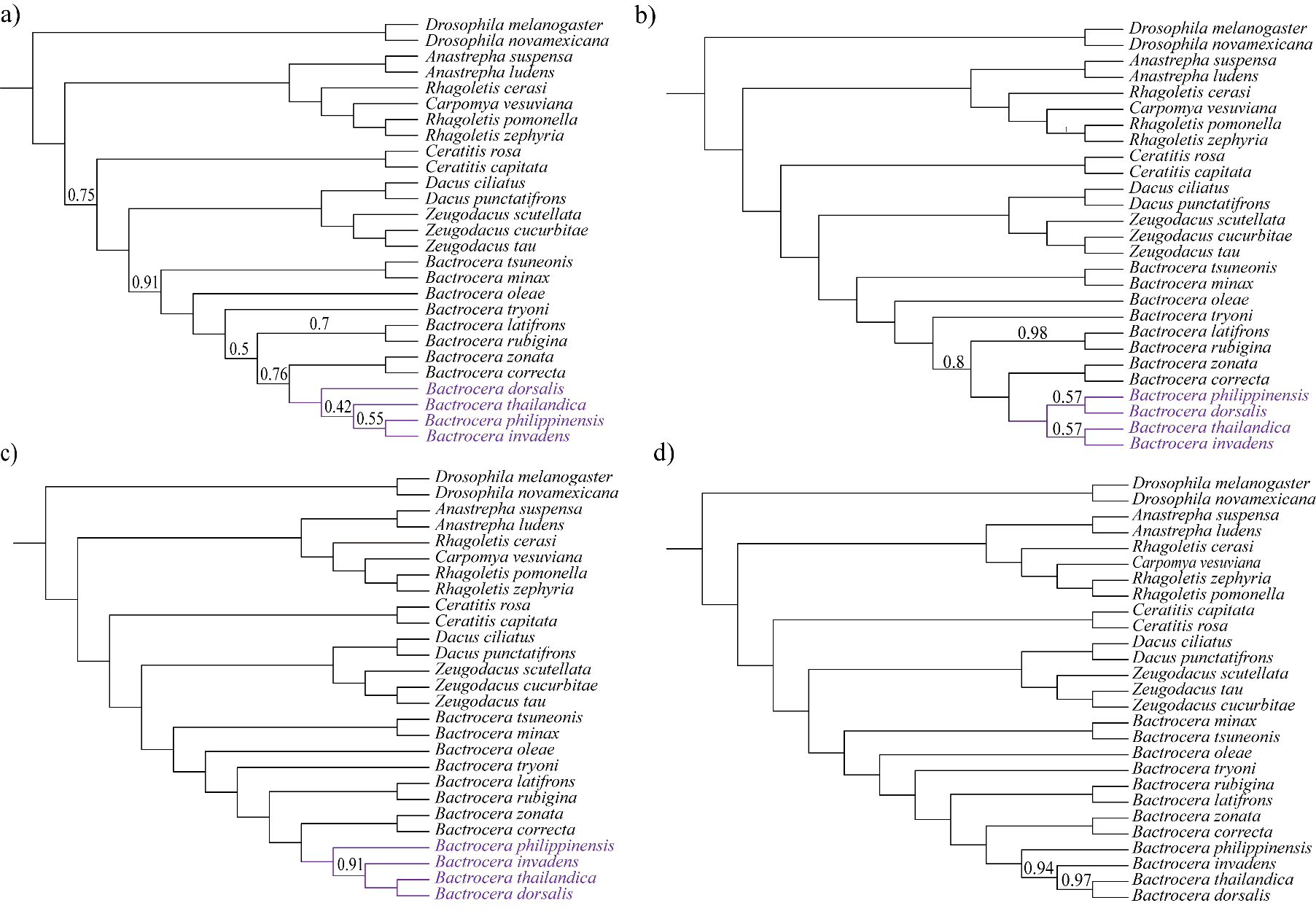


**Figture S4. Phylogeny tree of fruit flies estimated for BUSCO amino acid matrix of different taxon-occupancy datasets using a coalescent-based method.**

a) 100% taxon-occupancy BUSCO amino acid dataset, b) 90% taxon-occupancy BUSCO amino acid dataset, c) 75% taxon-occupancy BUSCO amino acid dataset, d) 50% taxon-occupancy BUSCO amino acid dataset. Increasing the number of loci tended to increase phylogenetic tree topological convergence and node support values. Colors highlight unstable topologies. Only support values smaller than 1 are shown.


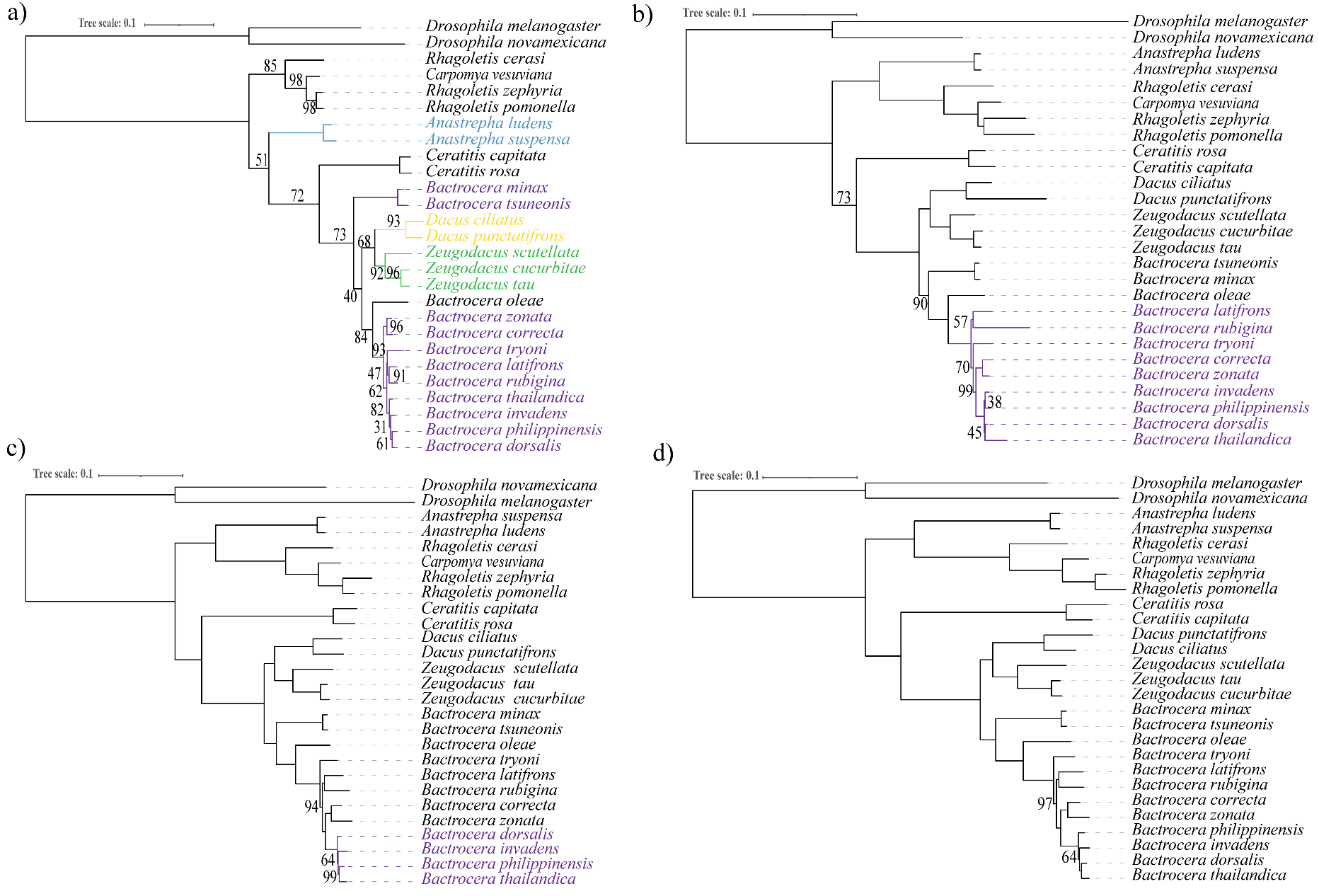


**Figture S5. Phylogeny tree of fruit flies estimated for AHE matrix of different taxon-occupancy datasets using a concatenation-based method.**

a) 100% taxon-occupancyAHE dataset, b) 90% taxon-occupancy AHE dataset, c) 75% taxon-occupancy AHE dataset, d) 50% taxon-occupancy AHE dataset. Increasing the number of loci tended to increase phylogenetic tree topological convergence and node support values. Colors highlight unstable topologies. Only support values smaller than 100 are shown.


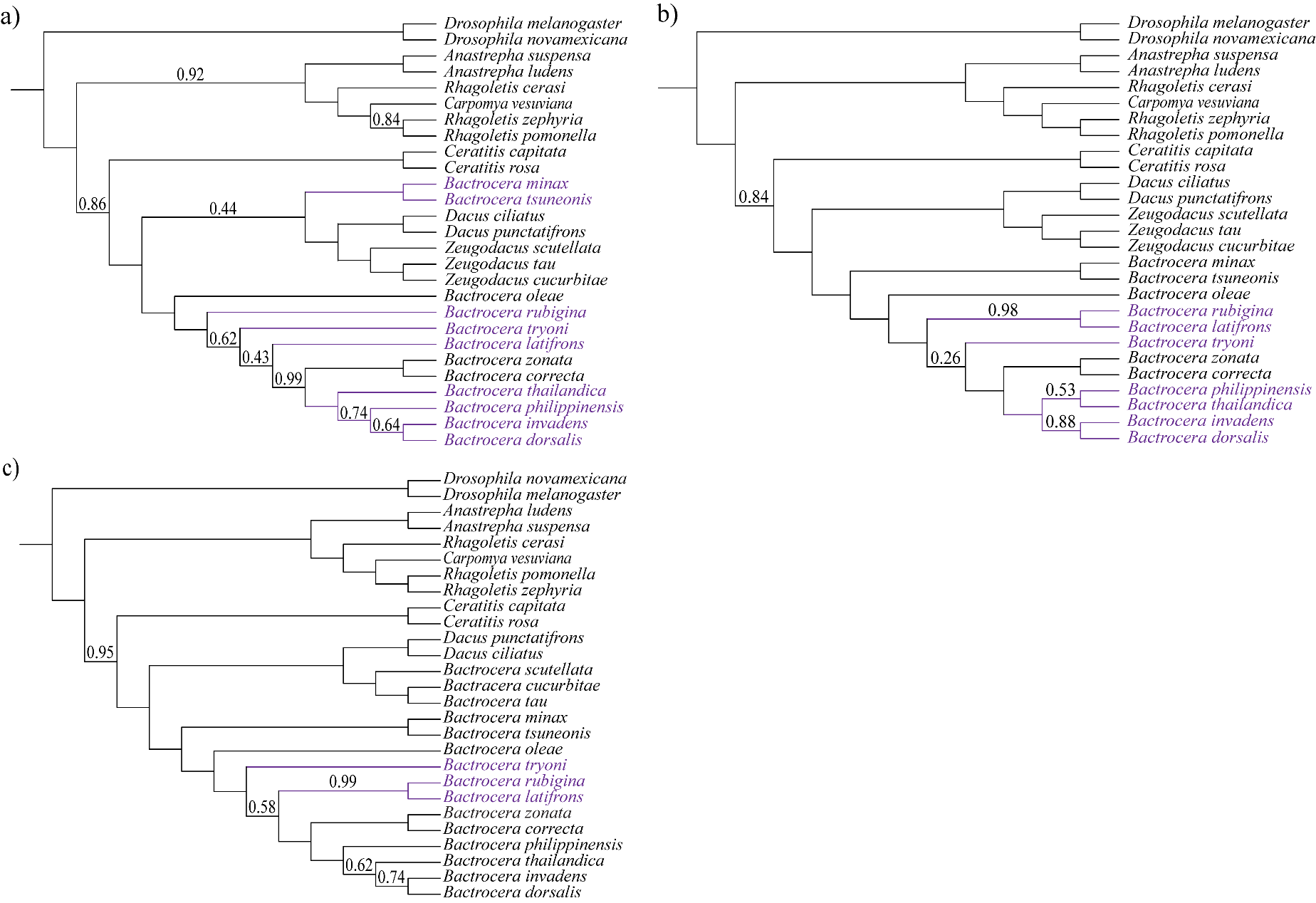


**Figture S6. Phylogeny tree of fruit flies estimated for AHE matrix of different taxon-occupancy datasets using a coalescent-based method.**

a) 90% taxon-occupancy AHE dataset, b) 75% taxon-occupancy AHE dataset, c)50% taxon-occupancy AHE dataset. Increasing the number of loci tended to increase phylogenetic tree topological convergence and node support values. Colors highlight unstable topologies. Only support values smaller than 1 are shown.


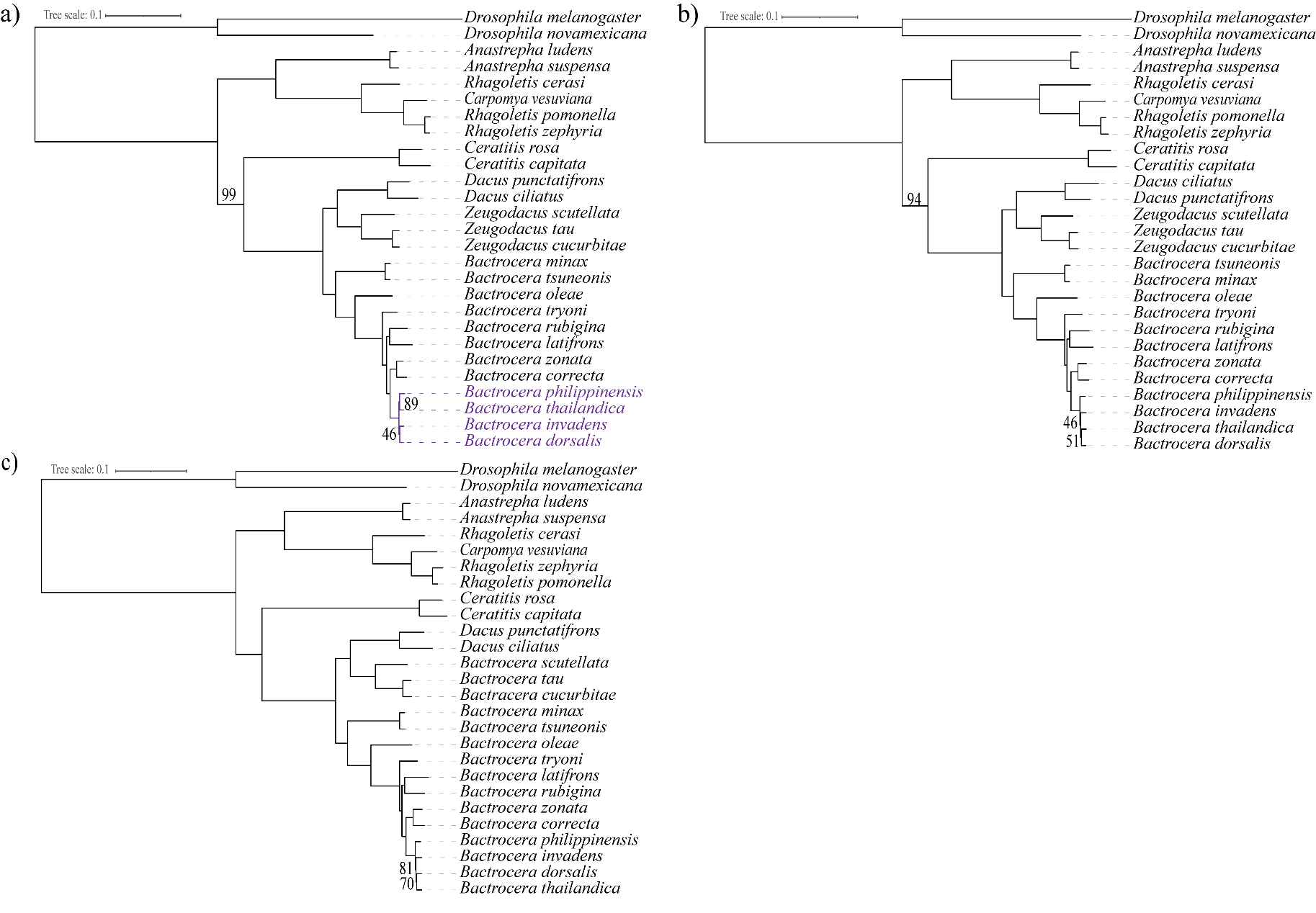


**Figture S7. Phylogeny tree of fruit flies estimated for UCE matrix of different taxon-occupancy datasets using a concatenation-based method.**

a) 90% taxon-occupancy UCE dataset, b) 75% taxon-occupancy UCE dataset, c) 50% taxon-occupancy UCE dataset. Increasing the number of loci tended to increase phylogenetic tree topological convergence and node support values. Only support values smaller than 100 are shown.


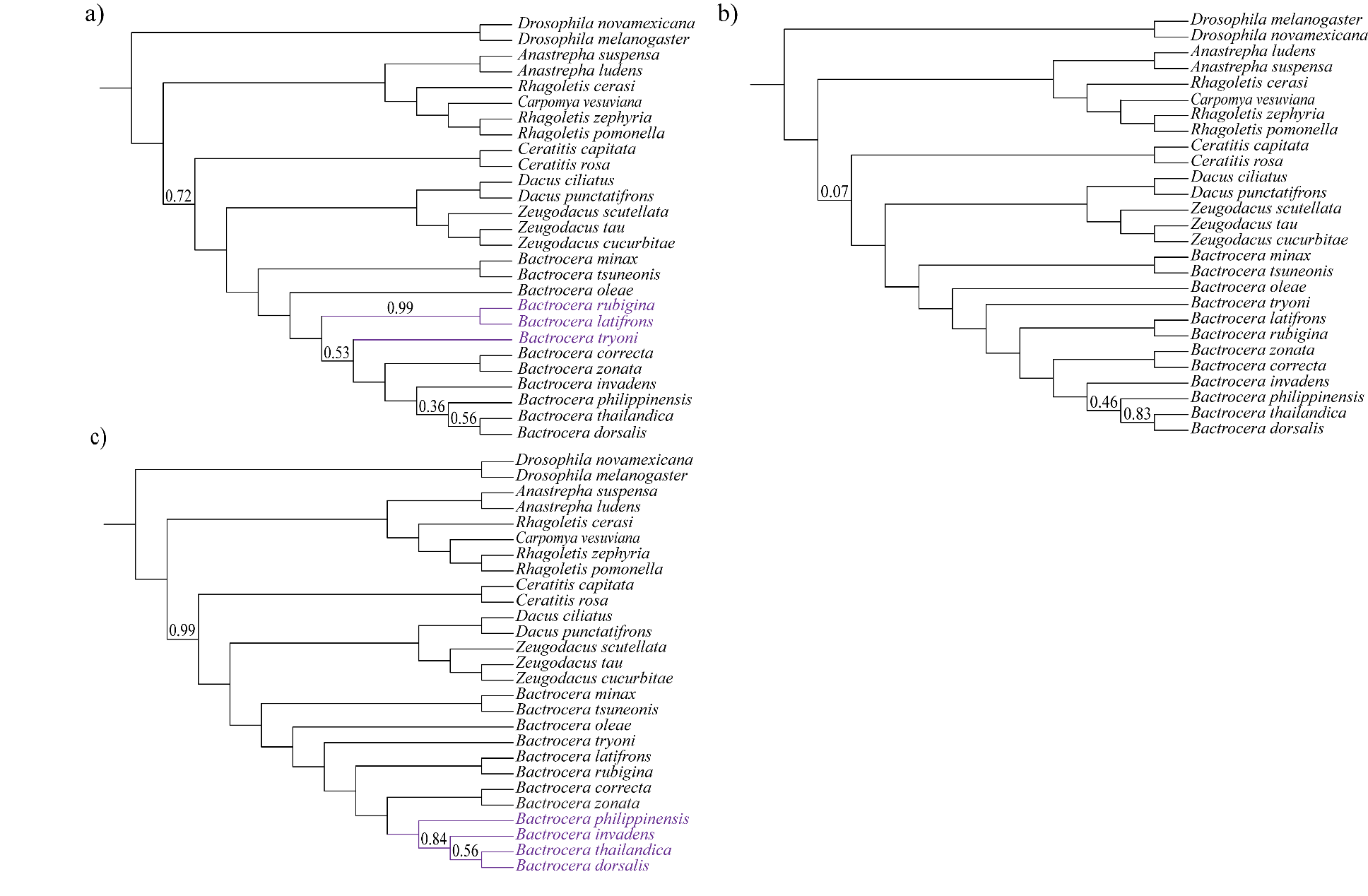


**Figture S8. Phylogeny tree of fruit flies estimated for UCE matrix of different taxon-occupancy datasets using a coalescent-based method.**

a) 90% taxon-occupancy UCE dataset, b) 75% taxon-occupancy UCE dataset, c) 50% taxon-occupancy UCE dataset. Increasing the number of loci tended to increase phylogenetic tree topological convergence and node support values. Only support values smaller than 1 are shown.


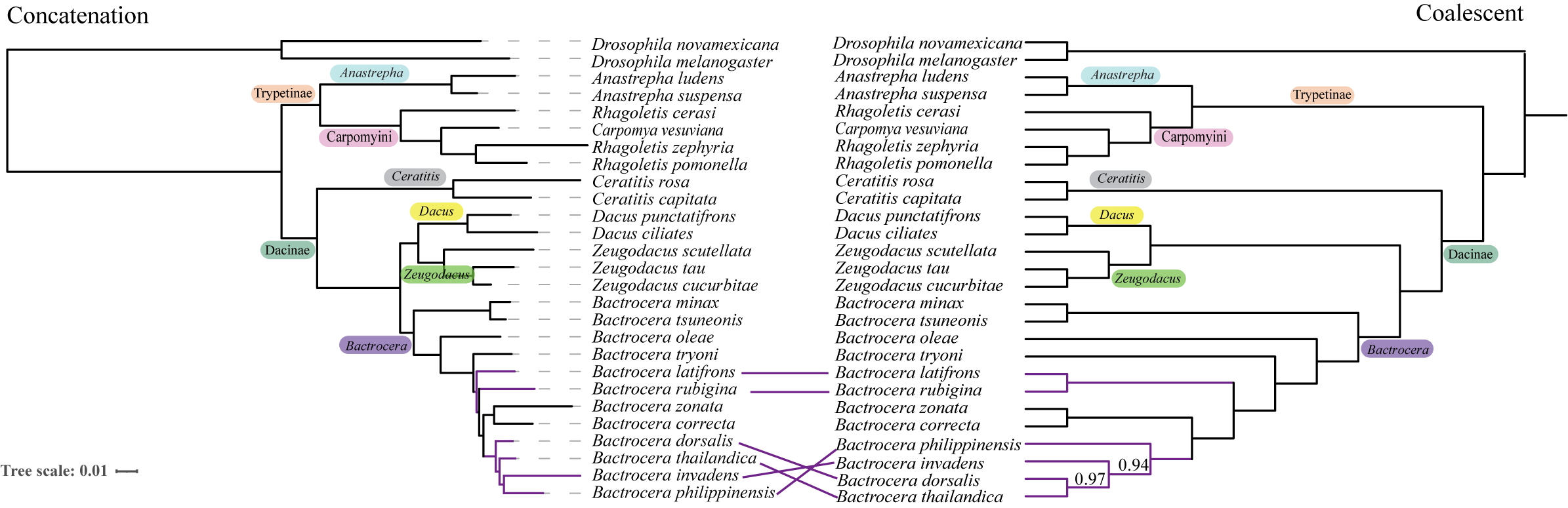


**Figture S9. Species trees for fruit flies in the Tephritidae family estimated for the BUSCO amino acid matrix of the 50% taxon-occupancy dataset.** Concatenation-based RAxML species phylogenetic tree (left) and coalescent-based ASTRAL species phylogenetic tree (right) were inferred by analysis of 1636 BUSCO loci. Branch support values denote bootstrap support and local posterior probability, respectively. Only support values smaller than 100% or 1 are shown.


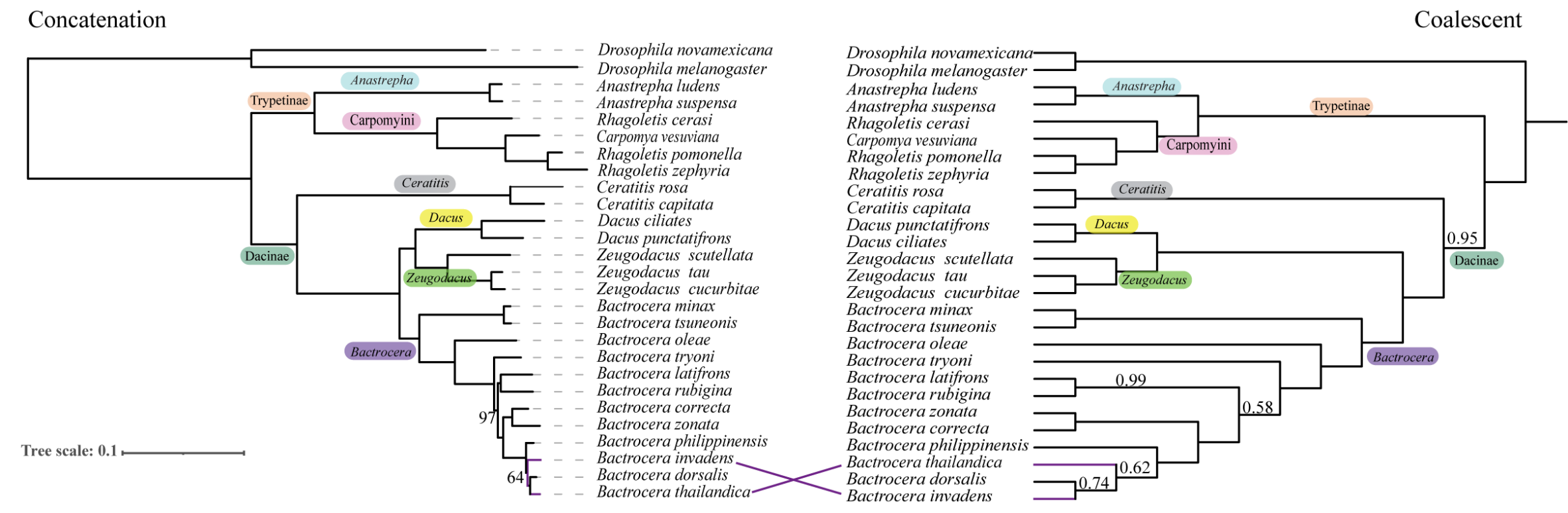


**Figture S10. Species trees for fruit flies in the Tephritidae family estimated for the AHE matrix of the 50% taxon-occupancy dataset.**

Concatenation-based RAxML species phylogenetic tree (left) and coalescent-based ASTRAL species phylogenetic tree (right) were inferred by analysis of 135 AHE loci. Branch support values denote bootstrap support and local posterior probability, respectively. Only support values smaller than 100% or 1 are shown.


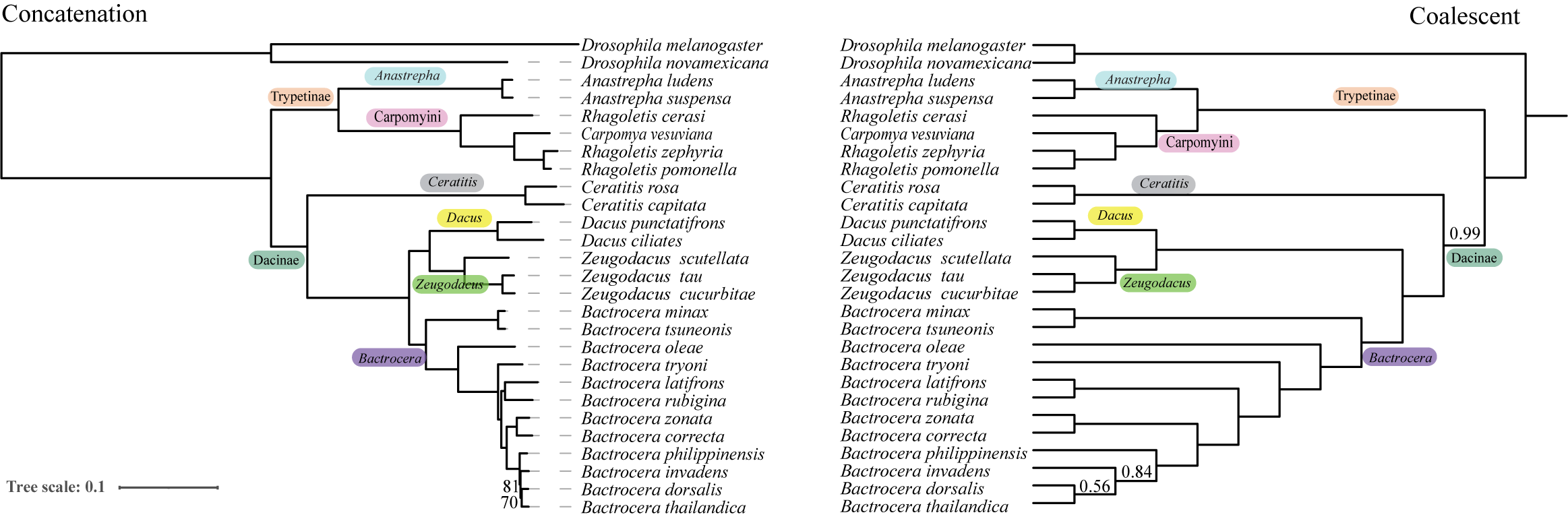


**Figture S11. Species trees for fruit flies in the Tephritidae family estimated for the UCE matrix of the 50% taxon-occupancy dataset.**

Concatenation-based RAxML species phylogenetic tree (left) and coalescent-based ASTRAL species phylogenetic tree (right) were inferred by analysis of 1327 UCE loci. Branch support values denote bootstrap support and local posterior probability, respectively. Only support values smaller than 100% or 1 are shown.


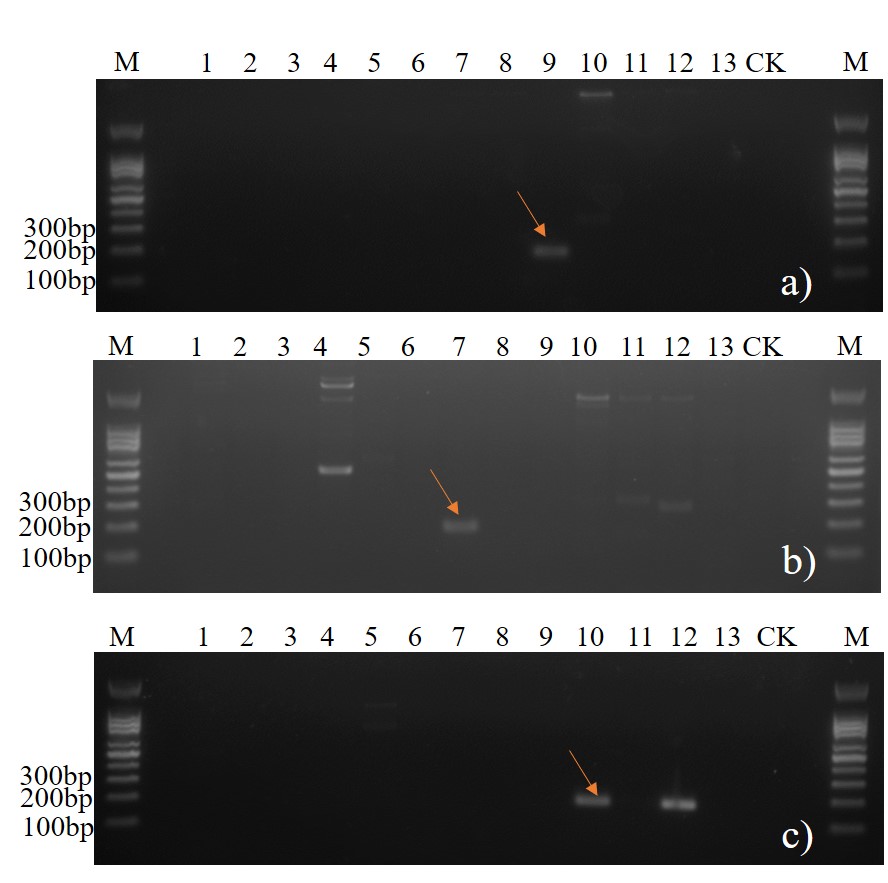


**Figture S12.** Electropherogram of the specific PCR amplification of species-specific sequence of fruit flies. a) Specific amplification based on the specific sequence Dpun6 in *D. punctatifrons*. b) Specific amplification based on the specific sequence Bcor7 in *B. correcta*. c) Specific amplification based on the specific sequence Asus3 in *A. suspensa*. M: 100bp DNA marker. Lane 1-13: DNA templates of *B. dorsalis*, *Z. tau*, *B. invadens*, *Z. cucurbitae*, *C. capitata*, *B. minax*, *B. correcta*, *B. oleae*, *D. punctatifrons*, *A. suspensa*, *B. latifrons*, *A. ludens*, *C. rosa*. CK: blank control.
